# Chemistry of NO_*x*_ and HNO_3_ Molecules with Gas‐Phase Hydrated O^.−^ and OH^−^ Ions

**DOI:** 10.1002/chem.202000322

**Published:** 2020-06-03

**Authors:** Jozef Lengyel, Milan Ončák, Martin K. Beyer

**Affiliations:** ^1^ Lehrstuhl für Physikalische Chemie Technische Universität München Lichtenbergstrasse 4 85748 Garching Germany; ^2^ Institut für Ionenphysik und Angewandte Physik Universität Innsbruck Technikerstrasse 25 6020 Innsbruck Austria

**Keywords:** atmospheric chemistry, heterogeneous reactions, hydration, nitric acid, nitric oxides

## Abstract

The gas‐phase reactions of O**^.^** ^−^(H_2_O)_*n*_ and OH^−^(H_2_O)_*n*_, *n=*20–38, with nitrogen‐containing atmospherically relevant molecules, namely NO_*x*_ and HNO_3_, are studied by Fourier transform ion cyclotron resonance (FT‐ICR) mass spectrometry and theoretically with the use of DFT calculations. Hydrated O**^.^** ^−^ anions oxidize NO**^.^** and NO_2_ **^.^** to NO_2_^−^ and NO_3_^−^ through a strongly exothermic reaction with enthalpy of −263±47 kJ mol^−1^ and −286±42 kJ mol^−1^, indicating a covalent bond formation. Comparison of the rate coefficients with collision models shows that the reactions are kinetically slow with 3.3 and 6.5 % collision efficiency. Reactions between hydrated OH^−^ anions and nitric oxides were not observed in the present experiment and are most likely thermodynamically hindered. In contrast, both hydrated anions are reactive toward HNO_3_ through proton transfer from nitric acid, yielding hydrated NO_3_^−^. Although HNO_3_ is efficiently picked‐up by the water clusters, forming (HNO_3_)_0–2_(H_2_O)_*m*_NO_3_^−^ clusters, the overall kinetics of nitrate formation are slow and correspond to an efficiency below 10 %. Combination of the measured reaction thermochemistry with literature values in thermochemical cycles yields Δ*H*_f_(O^−^(aq.))=48±42 kJ mol^−1^ and Δ*H*_f_(NO_2_^−^(aq.))=−125±63 kJ mol^−1^.

## Graphical abstract

**Solvation matters**: Hydrated O**^.^** ^−^ and OH^−^ ions in clusters are studied both experimentally and theoretically, revealing solvation effects on their ion chemistry. Hydration enables or hinders reactions with the most important nitrogen‐containing atmospheric molecules (see figure).

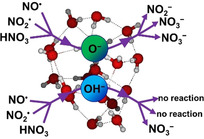

## Introduction

Heterogeneous reactions occurring on particle surfaces have attracted considerable attention owing to their environmental impact in several atmospheric processes. The most striking example is the conversion of reservoir species containing halogenated molecules on polar stratospheric clouds (PSCs) into photochemically active species that participate in catalytic ozone depletion.^1^ Atmospheric models predicted that more than 90 % of the ozone depletion is induced by chlorine activated in heterogeneous reactions on PSCs.^2^ In addition, heterogeneous chemistry has become a focus of interest for the conversion of atmospheric pollutants in the troposphere. Some atmospheric pollutants, such as nitrogen oxides (NO_*x*_), short for nitric oxide (NO) and nitrogen dioxide (NO_2_), exhibit efficient prooxidant activity in the reaction with OH**^.^** or O_3_.^3^ Nitrogen oxides are removed from the atmosphere either by dry or by wet deposition, mostly leading to HNO_3_.3a, 4 On the other hand, several reaction pathways have been suggested, in which nitric acid is recycled back to NO_*x*_ (known as renoxification) including reactions on aerosol surfaces^5^ and heterogeneous photochemistry.^6^ Therefore, the heterogeneous chemistry involving reactions in aerosol particles is believed to have a significant influence on the NO_*x*_/HNO_3_ balance of the atmosphere and greatly affects ozone concentration.5b, 7 The importance of HNO_3_ is also derived from the fact that PSCs of type I consist almost entirely of nitric acid hydrates.1a, 8

Atmospheric aerosols consist of neutrals and ions, volatile and nonvolatile molecules, liquid‐ and solid‐phase particles, and various impurities.^9^ The presence of impurities even at low concentrations dramatically influences aerosol properties and processes in the atmosphere. Particularly interesting is the influence of ions in atmospheric aerosol nucleation, enhancing the nucleation rates owing to the long‐range charge–dipole interactions between the core ions and the adsorbing polar molecules.^10^ This was invoked to explain the correlation between the global cloudiness and the intensity of cosmic radiation.^11^ Nowadays, the role of ions in aerosol formation is one of the most debated subjects in atmospheric chemistry.^12^

Cosmic radiation is the principal source of ionization and free electrons in the upper atmosphere. The chemistry of atmospheric anions is usually initiated by electron attachment to abundant molecules. In the gas phase, the key intermediates O_2_ **^.^** ^−^ and O**^.^** ^−^ are formed by the electron attachment to O_2_ and O_3_, respectively.^13^ The primary anions then undergo a cascade of ion–molecule reactions yielding NO_2_^−^, NO_3_^−^, and HSO_4_^−^ anions, which are among the most abundant anions in the troposphere and stratosphere. In these regions, uptake of single water molecules by anions is likely due to their high water affinity. Hydration might change the nature of reactions, dramatically affecting the electronic structure of transient anions. We have recently shown that electron attachment to HNO_3_/H_2_O particles predominantly lead to NO_3_^−^, which is in contrast to the dissociative electron attachment (DEA) to gas‐phase HNO_3_, yielding primarily NO_2_^−^.^14^ In addition, the hydration of the electron to form a hydrated electron and its reaction toward gas‐phase HNO_3_ lead to another reaction yielding OH^−^ and gaseous NO_2_.^15^ Gas‐phase reactions between HNO_3_ and hydrated anions are strongly influenced by the acid dissociation in water environment. For a series of hydrated ions, including O_2_ **^.^** ^−^(H_2_O)_*n*_ and CO_2_ **^.^** ^−^(H_2_O)_*n*_, *n=*31–70, the reactions of HNO_3_ provided direct evidence for proton transfer yielding NO_3_^−^.^16^ Similarly, the formation of NO_3_^−^ was observed in flow tube experiments even for the small oxygen hydrates, (H_2_O)_*n*≤3_X^−^ (X=O, OD, O_2_, DO_2_, O_3_).^17^ Particularly interesting chemistry was found in the reaction of O**^.^** ^−^(H_2_O)_*n*_, *n=*1–50, with HCl,^18^ in which a driving force of the reaction is proton transfer followed by evaporation of OH**^.^** from the cluster. However, in some cases OH**^.^** remains in the cluster until a second HCl molecule is picked up, resulting in Cl_2_ **^.^** ^−^(H_2_O)_*m*_ and additional water evaporation.

As reviewed,^19^ the ion–molecule reaction of strong acids (HNO_3_, HCl) with anions in water clusters proceeded exclusively by proton transfer. However, several types of reactions were investigated in the case of oxides, particularly oxidation and charge transfer. The effect of hydration on the reactivity of O**^.^** ^−^(H_2_O)_*n*_, *n=*0–2, was investigated toward several gaseous molecules in a temperature‐controlled fast flow reactor,^20^ in which the majority of molecules, namely CO, SO_2_, CH_4_, and N_2_O, followed the general trend with decreasing reactivity as the number of water molecules increased. In contrast, bare O**^.^** ^−^ did not exhibit any reactivity with O_2_ and CO_2_, but the oxidation reaction was enabled upon hydration yielding O_3_ **^.^** ^−^ and CO_3_ **^.^** ^−^. Particularly interesting was its reactivity toward NO**^.^** leading to NO_2_^−^, in which addition of one water ligand to O**^.^** ^−^ enhanced the reaction rate, but addition of the second water suppressed it. Nevertheless, the oxidation of NO**^.^** was found to proceed at a slightly higher degree of hydration with O**^.^** ^−^(H_2_O)_*n*_, *n*≤5.^21^ However, very little is known about reactions of O**^.^** ^−^ on larger water clusters. Only the formation of O**^.^** ^−^(H_2_O)_*n*_, *n=*0–59, itself was investigated by using a flow tube reactor.^22^ One of the reasons could be the instability of the O**^.^** ^−^ ion surrounded by water molecules and a subsequent formation of OH**^.^**OH^−^.^23^ Ab initio calculations have shown that, after sufficient hydration, both structures are energetically very close and most likely coexist.^24^ We have recently investigated the equilibrium between O**^.^** ^−^ and OH**^.^**OH^−^ structures in the water clusters by means of infrared multiple photon dissociation spectroscopy,^25^ in which evaporation of OH**^.^** was experimentally observed, indicating interconversion of O**^.^** ^−^ into OH**^.^**OH^−^.

In the present paper, we combine Fourier transform ion cyclotron resonance (FT‐ICR) mass spectrometry and density functional theory (DFT) calculations to find evidence for or against heterogeneous reactions between O**^.^** ^−^(H_2_O)_*n*_/OH^−^(H_2_O)_*n*_, *n=*20–38, anions, and nitrogen‐containing atmospherically relevant molecules, namely NO**^.^**, NO_2_ **^.^**, and HNO_3_. We report the rate coefficients and reaction enthalpies derived from the experimental data.

## Results and Discussion

The formation of the reactant ions, O**^.^** ^−^(H_2_O)_*n*_ and OH^−^(H_2_O)_*n*_, occurs in the reaction of anionic water clusters with N_2_O molecules. First, O**^.^** ^−^(H_2_O)_*n*_ cluster ions are formed as a primary product when the charge transfer occurs, yielding N_2_O^−^ followed by the formation of the oxygen radical anion (Reaction (1). The reaction is exothermic and leads to dissociation of water molecules. As recently shown,^25^, ^26^ the excess energy also induces an intracluster proton transfer reaction to yield OH**^.^**OH^−^(H_2_O)_*n*−1_, which can subsequently evaporate OH**^.^**, yielding OH^−^(H_2_O)_*n*−1_ (Reaction (2).

(1) $$\left(H{}_{2}O\right){}_{N}{}^{•}{}^{-}+N{}_{2}O\to O{}^{•}{}^{-}\left(H{}_{2}O\right){}_{n}+N{}_{2}+\left(N-n\right)H{}_{2}O$$

(2) $$O{}^{•}{}^{-}\left(H{}_{2}O\right){}_{n}\to \mathrm{OH}{}^{•}\mathrm{OH}{}^{-}\left(H{}_{2}O\right){}_{n-1}\to \mathrm{OH}{}^{-}\left(H{}_{2}O\right){}_{n-1}+\mathrm{OH}{}^{•}$$

**Figure 1:**
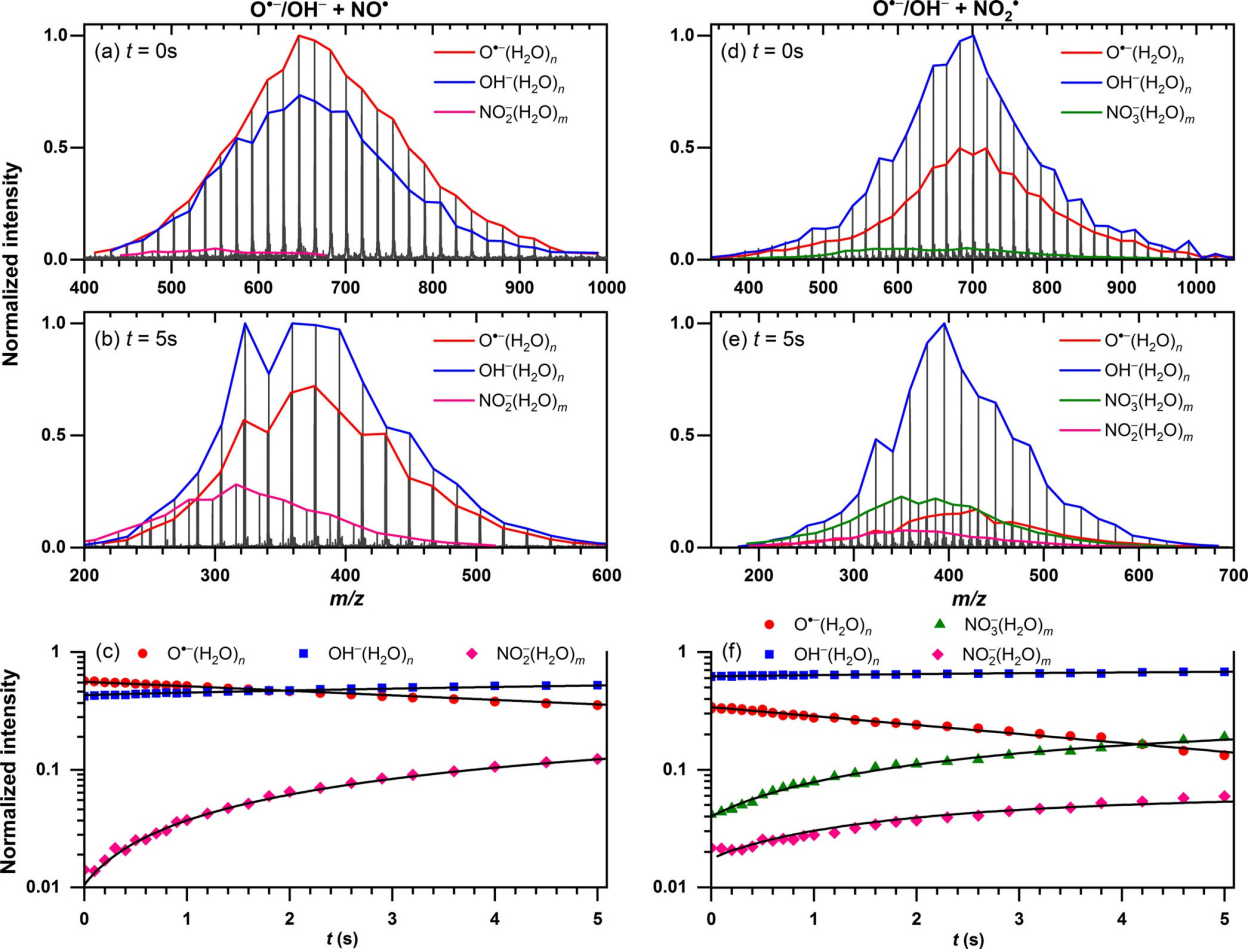
Mass spectra (a–d) and kinetic analysis (e–f) of the reaction of O**^.^** ^−^(H_2_O)_*n*_ and OH^−^(H_2_O)_*n*_ ions with NO_*x*_. Panels (a)–(c) show the reaction with NO**^.^** at a pressure of 4.0×10^−8^ mbar; panels (c)–(e) show the reaction with NO_2_ **^.^** at a pressure of 6.3×10^−8^ mbar.

The whole process is extremely fast, taking place within a few picoseconds,^23^ and occurs in the cluster source of the present experimental setup. The branching ratio between O**^.^** ^−^/OH^−^ ions can be controlled by tuning the source conditions such as laser power, timing of the laser pulse, N_2_O pressure in the pickup cell, opening of the piezovalve controlling the gas flow, etc. The present branching ratios are in the range from 4:3 to 2:3 for the O**^.^** ^−^ to OH^−^ ratio, as shown for initial conditions in Figure 1 a, d.

### Oxidation in the reaction of NO_x_ with O^.−^(H_2_O)_*n*_ ions

Although hydrated O**^.^** ^−^ ions show product formation with NO**^.^** and NO_2_ **^.^**, the hydrated OH^−^ ions are largely unreactive toward any of the NO_*x*_ species. Representative mass spectra for both reactions are displayed in Figure 1. Let us first discuss the reaction with NO**^.^**. At initial 0 s (Figure 1 a), the mass spectrum exhibits two intense cluster series, namely O**^.^** ^−^(H_2_O)_*n*_ and OH^−^(H_2_O)_*n*_ ions corresponding to the reactants with a branching ratio of approximately 4:3. Only traces of NO_2_^−^(H_2_O)_*n*_ ions below 2 % of the total intensity are found owing to reactive collisions during the ion accumulation (2 s) in the ICR cell. The intensity of the NO_2_^−^(H_2_O)_*n*_ ions increases with the reaction time and after 5 s (Figure 1 b), represents around 15 % of the total ion intensity. In parallel, intracluster reaction of O**^.^** ^−^(H_2_O)_*n*_ (Reaction 2) occurs with respective OH^−^ formation. A temporal evolution of ion intensities as a function of the reaction time is shown in Figure 1 c. The NO_2_^−^(H_2_O)_*n*_ is formed either by oxidation of NO**^.^** by O**^.^** ^−^(H_2_O)_*n*_ (Reaction (3), or through radical–radical recombination of NO**^.^** with OH**^.^** followed by proton transfer (Reaction 3′), as O**^.^** ^−^(H_2_O)_*n*_ is in equilibrium with OH^−^(OH**^.^**)(H_2_O)_*n*−1_.^25^ Both mechanistic pathways lead to the same product. At longer times, the cluster size distribution shifts to smaller sizes owing to loss of water molecules upon blackbody infrared radiative dissociation (BIRD). Neither formation of HONO**^.^** ^−^(H_2_O)_*n*_ ions nor loss of ion intensity of OH^−^(H_2_O)_*n*_ cluster ions are observed. Therefore, there is no evidence for the reaction between NO**^.^** and OH^−^(H_2_O)_*n*_ (Reaction (4).

(3) $$O{}^{•}{}^{-}\left(H{}_{2}O\right){}_{n}+\mathrm{NO}{}^{•}\to \mathrm{NO}{}_{2}{}^{-}\left(H{}_{2}O\right){}_{m}+\left(n-m\right)H{}_{2}O$$

(3′) $$\mathrm{OH}{}^{-}\left(\mathrm{OH}{}^{•}\right)\left(H{}_{2}O\right){}_{n-1}+\mathrm{NO}{}^{•}\to \mathrm{NO}{}_{2}{}^{-}\left(H{}_{2}O\right){}_{m}+\left(n-m\right)H{}_{2}O$$

(4) $$\mathrm{OH}{}^{-}\left(H{}_{2}O\right){}_{n}+\mathrm{NO}{}^{•}\to \mathrm{NO}{}^{•}\mathrm{OH}{}^{-}\left(H{}_{2}O\right){}_{n}\;\left(\mathrm{not}\;\mathrm{observed}\right)$$

To determine the reaction rate, the time evolution of ion intensities is fitted according to pseudo‐first‐order kinetics. Reaction (3) proceeds with *k*_abs_(3)=5.0±1.5×10^−11^ cm^3^ s^−1^. The measured experimental rate coefficient *k*_abs_(3) is compared with the calculated collision rates to determine the reaction efficiency. The collision rates for *n=*25 are estimated as *k*_HSA_=9.9×10^−10^ cm^3^ s^−1^, *k*_SCC_=2.0×10^−9^ cm^3^ s^−1^, resulting in a low efficiency of 3.3 %.

The plot of mean cluster sizes as a function of time, see Figure S1(b) in the Supporting Information, shows a significant shift in ion distribution of NO_2_^−^(H_2_O)_*m*_ to smaller cluster sizes relative to that of the O**^.^** ^−^(H_2_O)_*n*_. The loss of water molecules indicates an exothermic reaction. We therefore applied nanocalorimetry, in which the thermochemistry of the reaction is determined from the average number of evaporated water molecules. A nanocalorimetric fit reveals that the oxidation leads to the evaporation of 6.1±1.1 water molecules, which corresponds to a strongly exothermic reaction with an enthalpy of Δ_r_ *H*_exp,298K_(3)=−263±47 kJ mol^−1^.

**Figure 2:**
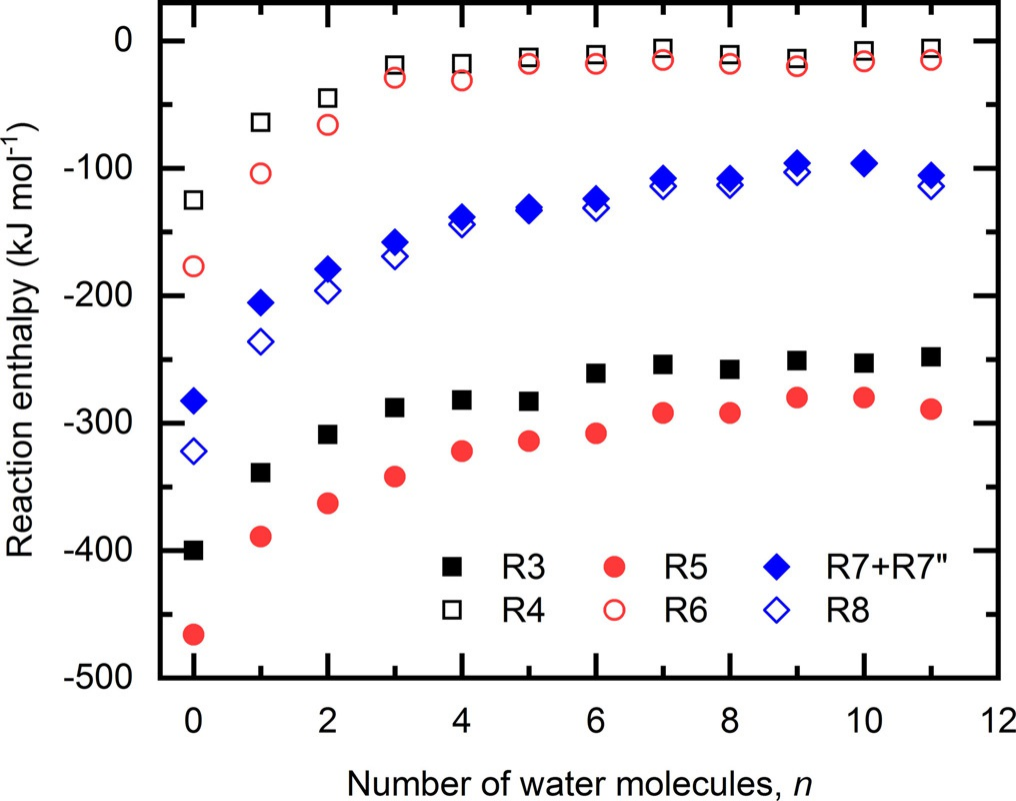
Calculated reaction enthalpies depending on the number of water molecules for *n*=*m* in the respective equations. Full/empty symbols represent reaction of O**^.^** ^−^/OH^−^ with NO**^.^** (black squares), NO_2_ **^.^** (red circles), and HNO_3_ (blue diamonds).

The high exothermicity of reaction (3) is a consequence of covalent bond formation between O**^.^** ^−^ and NO**^.^**. The evolution of energies with the increasing number of water molecules is shown in Figure 2. In the gas phase, the enthalpy of reaction (3) is calculated to be −400 kJ mol^−1^, this value is, however, lowered by the higher hydration energy of O**^.^** ^−^ compared with NO_2_^−^. The average hydration enthalpies for a low number of water molecules differ substantially, calculated to be −99 and −70 kJ mol^−1^ for O**^.^** ^−^ and NO_2_^−^, respectively, hydrated by one to four water molecules. The calculated average enthalpy of −253 kJ mol^−1^ for reaction (3) with 7–11 water molecules agrees within error limits with the experimentally obtained value (Table 1).

**Table 1:** Reaction kinetics and energetics for reactions of NO_*x*_ and HNO_3_ with O**^.^** ^−^(H_2_O)_*n*_ and OH^−^(H_2_O)_*n*_ ions. Enthalpies calculated for *n*=*m* at the B3LYP+D2/TZVP level, Δ_r_ *H*_theo_, are shown as averages for structures solvated by 7–11 water molecules along with standard deviation. See the Supporting Information for the respective structures.

Reaction	Reaction kinetics				Reaction energetics		
	*k*_exp_ [10^−10^ cm^3^ s^−1^]	*k*_HSA_ [10^−10^ cm^3^ s^−1^]	*k*_SCC_ [10^−10^ cm^3^ s^−1^]	*Φ* [%]	*N*	Δ_r_ *H*_exp_ [kJ mol^−1^]	Δ_r_ *H*_theo_ [kJ mol^−1^]
(3)	0.50±0.15	9.89	20.3	3.3	6.1	−263±47^[a]^	−253±4
(4)							−9±3
(5)	0.93±0.28	9.52	19.5	6.5	6.6	−286±42^[a]^	−286±6
(6)							−17±2
(7)+(7′′)	2.0±0.6	17.6	31.4	8.2		−80±42^[b]^	−103±6
(8)	2.3±0.7	17.6	31.4	9.4		−82.5^[c]^	−108±8

[a] This work. [b] Literature value for aqueous solution29a and this work. [c] Literature value for aqueous solution.29a

Interestingly, reaction (4) is predicted to be markedly exothermic for a low number of water molecules (Figure 2), with a reaction enthalpy of −125 kJ mol^−1^ for the reaction in the gas phase, forming an ON⋅⋅⋅OH^−^ complex with a distance between both moieties of 1.86 Å. Differences in hydration enthalpy of OH^−^ and [HONO]^−^, however, lower this value to −9 kJ mol^−1^ for 7–11 water molecules (Table 1). This low value is comparable to the NO**^.^** hydration enthalpy on water clusters (−7 kJ mol^−1^ for 1–11 water molecules) and explains why the reaction is not observed in the experiment.

The reactions with NO_2_ **^.^** as a neutral reactant occur in a similar fashion to that of NO**^.^** molecules, in which oxidation of NO_2_ **^.^** by O**^.^** ^−^ is observed (Reaction (5)). Again, if O**^.^** ^−^(H_2_O)_*n*_ rearranges to OH^−^(OH**^.^**)(H_2_O)_*n*−1_, radical–radical recombination followed by proton transfer yields the same product (Reaction (5′)). The hydrated OH^−^ ion is largely unreactive, with an upper limit for the rate of *k*_abs_<10^−12^ cm^3^ s^−1^. The representative mass spectra are shown in Figure 1 d, e. The mass spectrum taken at 0 s (Figure 1 d) is dominated by both reactant ions with a branching ration 3:2 for OH^−^, however, already at the initial time, one can observe a small amount (≈5 %) of NO_3_^−^ as a product ion. Traces of NO_2_^−^ are also observed, which are assigned to NO**^.^** as an impurity in the NO_2_ **^.^** gas bottle. Temporal evolution of the ion intensities with the reaction time is shown in Figure 1 f. After 4 s of reaction, the abundances of reactant O**^.^** ^−^ ions and product NO_3_^−^ ions are almost exactly equal. In the further course of reaction, the abundance of NO_3_^−^ increases until the O**^.^** ^−^ ion intensity disappears.

(5) $$O{}^{•}{}^{-}\left(H{}_{2}O\right){}_{n}+\mathrm{NO}{}_{2}{}^{•}\to \mathrm{NO}{}_{3}{}^{-}\left(H{}_{2}O\right){}_{m}+\left(n-m\right)H{}_{2}O$$

(5′) $$\mathrm{OH}{}^{-}\left(\mathrm{OH}{}^{•}\right)\left(H{}_{2}O\right){}_{n-1}+\mathrm{NO}{}_{2}{}^{•}\to \mathrm{NO}{}_{3}{}^{-}\left(H{}_{2}O\right){}_{m}+\left(n-m\right)H{}_{2}O$$

(6) $$\mathrm{OH}{}^{-}\left(H{}_{2}O\right){}_{n}+\mathrm{NO}{}_{2}{}^{•}\to \mathrm{NO}{}_{2}{}^{•}\mathrm{OH}{}^{-}\left(H{}_{2}O\right){}_{n}\;\left(\mathrm{not}\;\mathrm{observed}\right)$$

Reaction (5) proceeds with a rate coefficient of *k*_abs_(5)=9.3±2.8×10^−11^ cm^3^ s^−1^. In comparison with the calculated collision rates (Table 1), the reaction efficiency reaches around 6.5 %. By counting the number of evaporating water molecules by using nanocalorimetry, the NO_2_ oxidation reveals strong exothermicity with an enthalpy of Δ_r_ *H*_exp,298K_(5)=−286±42 kJ mol^−1^.

The calculated average reaction enthalpy of reaction (5) is −286 kJ mol^−1^ for 7–11 water molecules, again in perfect agreement with the experiment. Similarly as for reaction (3), there is a very high reaction enthalpy in the gas phase (−466 kJ mol^−1^), which is reduced upon hydration owing to the difference in the solvation energies of O**^.^** ^−^ and NO_3_^−^. The computation reveals reaction (6) to be only mildly exothermic with the average enthalpy of −17 kJ mol^−1^, similarly to the calculated adsorption enthalpy of NO_2_ **^.^** on a water clusters (−12 kJ mol^−1^ for 1–11 water molecules). In the gas phase, the formed OH^−^⋅⋅⋅NO_2_ moiety with the O–N distance of 2.24 Å is stable (−177 kJ mol^−1^), the relative hydration energy of OH^−^ and the [OH⋅⋅⋅NO_2_]^−^ moiety makes the reaction significantly less exothermic.

Although both reactions are accompanied by high exothermicity, they are quite inefficient, occurring at 3.3 % of the collisions for NO and 6.5 % for NO_2_. Apparently, the low reaction efficiency might be a consequence of two complementary processes: (*i*) NO_*x*_ hydrophobicity towards water clusters and (*ii*) suppression of O**^.^** ^−^ reactivity upon hydration. A significantly smaller sticking coefficient for collisions of NO_*x*_ with large water clusters compared with hydrophilic molecules like water or methanol was found in pick‐up experiments.^27^ For instance, the values reported by Ahmed et al.27a showed that the sticking coefficients of NO**^.^** and NO_2_ **^.^** are smaller by factors of 11 and 20 compared with methanol, indicating a very low pick‐up efficiency. An additional effect might be the influence of hydration on the reaction between NO_*x*_ and hydrated O**^.^** ^−^ ions, as observed in a fast flow reactor.^20^, ^21^ In these experiments, a decreasing reactivity as a function of cluster size was observed. Viggiano et al.^20^ reported that the rate coefficients decreased two times upon addition of one water molecule to the O**^.^** ^−^(H_2_O) cluster. However, although the number of water molecules affects O**^.^** ^−^(H_2_O)_*n*_ reactivity with *n*≤2, no such influence was found in the range of *n=*2–5 and the rate coefficients were around 1.8×10^−10^ cm^3^ s^−1^, representing about 30 % reaction efficiency.^21^ Our experimental rate coefficient for O**^.^** ^−^+NO**^.^** in large water clusters is about a factor of 3.6 lower than the rate coefficients reported for small ones. Our observations indicate that the uptake of NO_*x*_ onto the aerosol particles and their subsequent oxidation on the particle surface do not significantly contribute to the formation of the atmospheric NO_3_^−^, which is rather formed through gas‐phase oxidation of NO_*x*_ yielding gaseous HNO_3_,3a followed by its uptake onto the aerosol particles.

To clarify the mechanism of the intracluster reaction, we have carried out molecular dynamics simulations and optimized structures with NO**^.^** and NO_2_ **^.^** on different positions with respect to the O**^.^** ^−^(H_2_O)_*n*_ cluster. We have found that the impacting radicals react only when the orientation favors the direct contact with the O**^.^** ^−^ anion. Otherwise, they might stay on the surface and eventually react. The adsorption energy, however, is small, on neat water. We calculated the average adsorption enthalpy on (H_2_O)_*n*_, *n=*7–11, as −7 kJ mol^−1^ and −12 kJ mol^−1^ for NO**^.^** and NO_2_ **^.^**, respectively. Our calculations thus corroborate the low rate coefficient measured in the experiment in a qualitative way.

We have previously shown that the thermochemistry derived from our cluster studies is compatible with bulk aqueous solutions.^15^, ^28^ The enthalpy of reaction (5) combined with literature thermochemistry^29^ yields the heat of formation of the O**^.^** ^−^ radical in aqueous solution of Δ*H*_f_(O**^.^** ^−^(aq.)) = 48 ± 42 kJ mol^−1^, see the Supporting Information section S2.2 for details. Combining this value with literature thermochemistry and the enthalpy of reaction (3) measured here yields the heat of formation of the nitrite ion in aqueous solution, Δ*H*_f_(NO_2_^−^(aq.))=−125±63 kJ mol^−1^.

### Proton transfer in the reaction of HNO_3_ with O^.−^(H_2_O)_*n*_ and OH^−^(H_2_O)_*n*_ ions

**Figure 3:**
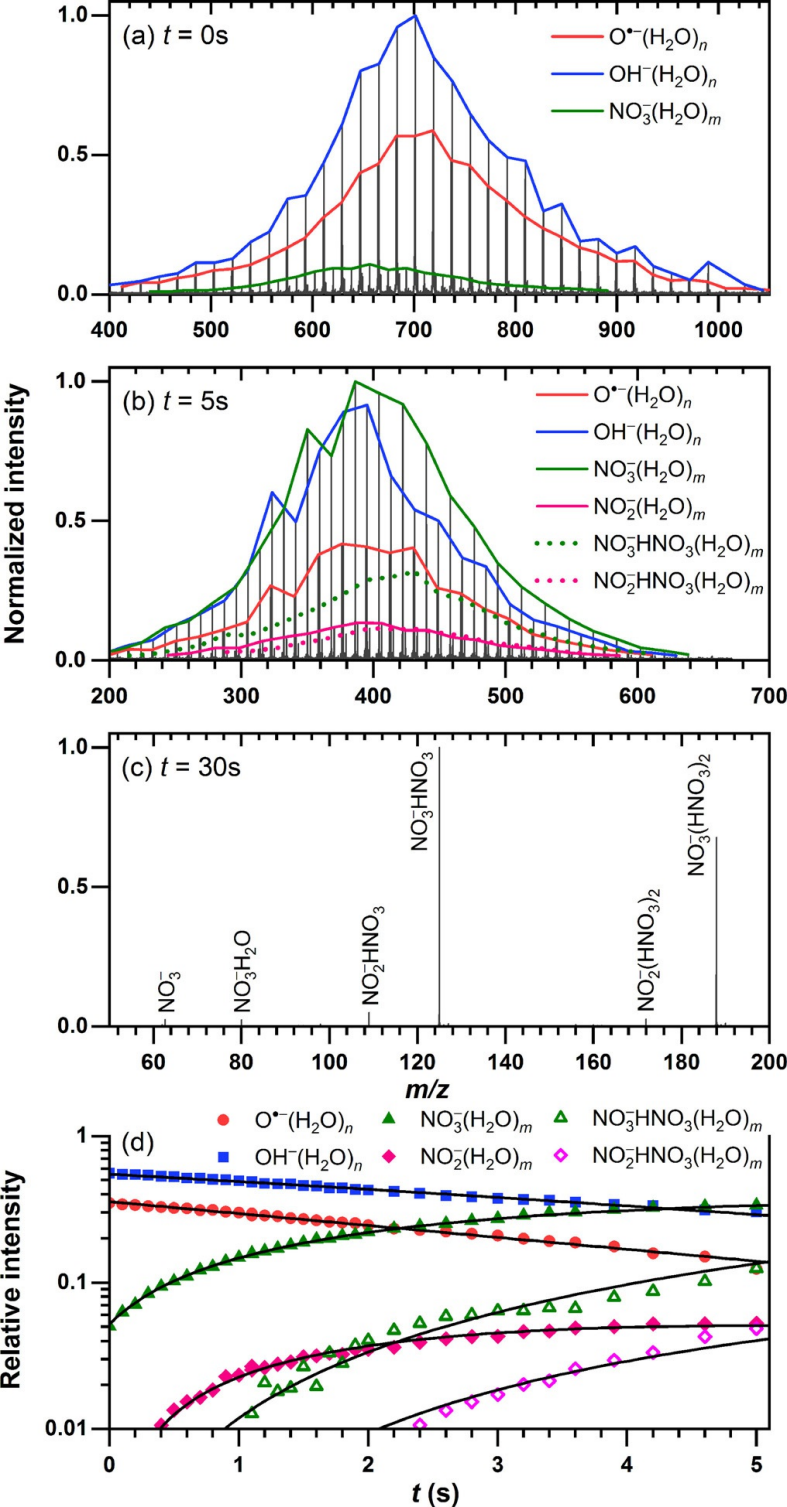
Mass spectra at different reaction times (a–c) and kinetic analysis (d) of the reaction of O**^.^** ^−^(H_2_O)_*n*_ and OH^−^(H_2_O)_*n*_ ions with HNO_3_ at a pressure of 5.4×10^−8^ mbar. Panel (d) represents the pseudo‐first‐order kinetic fit of O**^.^** ^−^(H_2_O)_*n*_ (blue squares), OH^−^(H_2_O)_*n*_ (red circles), NO_3_^−^(H_2_O)_*n*_ (green triangles), NO_3_^−^HNO_3_(H_2_O)_*n*_ (green open triangles), NO_2_^−^(H_2_O)_*n*_ (pink diamonds), and NO_2_^−^HNO_3_(H_2_O)_*n*_ (pink open diamonds).

In contrast to NO_*x*_ molecules, gaseous HNO_3_ reacts with both O**^.^** ^−^(H_2_O)_*n*_ and OH^−^(H_2_O)_*n*_, *n=*22–38. The respective mass spectra are shown in Figure 3. At first glance, experiments with HNO_3_ exhibit higher complexity than in the previous cases with NO_*x*_ molecules. The reaction results in intense NO_3_^−^(H_2_O)_*m*_ formation, but a very small abundance (<2 %) of NO_3_^−^(OH**^.^**)(H_2_O)_*m*_ is also observed. Traces of NO_2_^−^(H_2_O)_*m*_ are present owing to decomposition of HNO_3_ on the apparatus walls, which leads to HONO. Figure 3 a exemplifies an initial distribution of clusters at a nominal time of 0 s, in which a small amount of 5 % of NO_3_^−^ as a product ion is already present owing to reactions occurring during the accumulation process. The results indicate that the primary reaction is proton transfer to hydrated O**^.^** ^−^ and OH^−^ ions, yielding NO_3_^−^ (Reactions (7), (7′), and (8)). The NO_3_^−^(OH**^.^**)(H_2_O)_*m*_ product at some point loses OH**^.^** (Reaction (7′′)). After 5 s of reaction time (Figure 3), the NO_3_^−^ moiety dominates the mass spectrum and uptake of additional HNO_3_ is also found (Reaction (9)). At longer times (Figure 3 c), multiple pick‐up of HNO_3_ and the significant effect of BIRD take place, leading to complete water evaporation and the formation of NO_3_^−^HNO_3_ and NO_3_^−^(HNO_3_)_2_ cluster ions.

(7) $$O{}^{•}{}^{-}\left(H{}_{2}O\right){}_{n}+\mathrm{HNO}{}_{3}\to \mathrm{NO}{}_{3}{}^{-}\left(\mathrm{OH}{}^{•}\right)\left(H{}_{2}O\right){}_{m}+\left(n-m\right)H{}_{2}O$$

(7′) $$\mathrm{OH}{}^{-}\left(\mathrm{OH}{}^{•}\right)\left(H{}_{2}O\right){}_{n-1}+\mathrm{HNO}{}_{3}\to \;\;\;\;\;\;\;\;\;\mathrm{NO}{}_{3}{}^{-}\left(\mathrm{OH}{}^{\cdot }\right)\left(H{}_{2}O\right){}_{m}+\left(n-m\right)H{}_{2}O$$

(7′′) $$\mathrm{NO}{}_{3}{}^{-}\left(\mathrm{OH}{}^{•}\right)\left(H{}_{2}O\right){}_{m}\to \mathrm{NO}{}_{3}{}^{-}\left(H{}_{2}O\right){}_{m}+\mathrm{OH}{}^{•}$$

(8) $$\mathrm{OH}{}^{-}\left(H{}_{2}O\right){}_{n}+\mathrm{HNO}{}_{3}\to \mathrm{NO}{}_{3}{}^{-}\left(H{}_{2}O\right){}_{m}+\left(n-m+1\right)H{}_{2}O$$

(9) $$\mathrm{NO}{}_{3}{}^{-}\left(H{}_{2}O\right){}_{m}+\mathrm{HNO}{}_{3}\to \mathrm{NO}{}_{3}{}^{-}\mathrm{HNO}{}_{3}\left(H{}_{2}O\right){}_{l}+\left(m-l\right)H{}_{2}O$$

(10) $$\mathrm{NO}{}_{3}{}^{-}\mathrm{HNO}{}_{3}\left(H{}_{2}O\right){}_{l}+\mathrm{HNO}{}_{3}\to \;\;\;\;\;\;\;\;\;\mathrm{NO}{}_{3}{}^{-}\left(\mathrm{HNO}{}_{3}\right){}_{2}\left(H{}_{2}O\right){}_{k}+\left(l-k\right)H{}_{2}O$$

The kinetic analysis is complicated by several effects: reactions (7′′) and (8) lead to the same product; OH**^.^** radicals statistically evaporate from the product of reaction (7) or (7′), leading to hydrated nitrate with a delay that depends on cluster size; reactions (9) and (10) lead to products of the same nominal mass as OH^−^(H_2_O)_*n*_ and NO_3_^−^(H_2_O)_*m*_, respectively, which are only partially resolved. Secondary reactions analogous to (9) and (10) may occur while the OH**^.^** is still present. The relative intensities extracted for the fit in Figure 3 d are therefore associated with significant uncertainties. To get an estimate for the rate of HNO_3_ uptake by the clusters, we fitted the data assuming pseudo‐first‐order kinetics, and by combining reactions (7)/(7′) and (7′′) into a single step. In other words, all OH**^.^**‐containing products are neglected in the fit, as their intensity cannot be extracted from the data. As an aqueous 70 % HNO_3_ solution is used in the experiment, which is close to the azeotropic point, we assume that the composition of the binary HNO_3_/H_2_O mixture in the gas phase is the same as in solution. Therefore, the partial pressure of HNO_3_ is estimated as 70 % of the total measured pressure. Then, we obtain the rate coefficients *k*_abs_(7+7′′)=(2.0±0.6)×10^−10^ cm^3^ s^−1^ and *k*_abs_(8)=(2.3±0.7)×10^−10^ cm^3^ s^−1^, which correspond to 8.2 % and 9.4 % collisional efficiency, respectively.

The reaction efficiency for HNO_3_ is about a factor of 2.5 greater than for NO**^.^**, in line with the relative sticking efficiencies determined by Whitehead and co‐workers27a where the ratio of the HNO_3_ to NO**^.^** coefficients is around 3, albeit for neutral water clusters. A significant discrepancy between our reaction efficiency and sticking efficiency is found only when the reaction with HNO_3_ is compared to NO_2_ **^.^**. Although the adsorption efficiency of HNO_3_ to water cluster is almost six times larger when compared with NO_2_ **^.^**, the ratio of reaction efficiencies of the HNO_3_ to NO_2_ **^.^** in our clusters is only approximately 1.2. The most straightforward explanation is that in the present experiment, a chemical reaction occurs between the incoming neutral molecule and the charged reactive species in the water cluster, and this changes the nature of the event completely, compared with neat neutral water clusters.

Both the net reactions (7+7′′) and (8) are exothermic, with reaction enthalpies of Δ_r_ *H*_exp_(7+7′′)=−80±42 kJ mol^−1^ and Δ_r_ *H*_exp_(8)=−82.5 kJ mol^−1^, derived from literature thermochemistry for bulk aqueous solution29a and the measured value of Δ*H*_f_(O**^.^** ^−^(aq.)), see the Supporting Information section S2.2 for details. These experimental values compare favorably with our cluster calculations, which yield Δ_r_ *H*_theo_(7+7′′)=−103 and Δ_r_ *H*_theo_(8)=−108 kJ mol^−1^, respectively (Table 1). Association of nitric acid molecules, (9) and (10), also proceeds exothermically, with the enthalpies of −112 and −81 kJ mol^−1^, respectively. In addition, our calculations reveal that, for more than four water molecules, the structure of both NO_3_^−^HNO_3_(H_2_O)_*k*_ and NO_3_^−^(HNO_3_)_2_(H_2_O)_*k*_ clusters consist of two NO_3_^−^ and one H_3_O^+^ ions for up to 11 water molecules considered.

## Conclusion

The hydrated O**^.^** ^−^ oxidizes NO**^.^** or NO_2_ **^.^** to NO_2_^−^ and NO_3_^−^, whereas OH^−^ is found to be unreactive to NO_*x*_. DFT calculations indicate that OH^−^ reactivity toward NO_*x*_ is thermodynamically hindered, whereas the oxidation reaction with O**^.^** ^−^ is strongly exothermic. Despite high heat release owing to a covalent bond formation, the oxidation has slow kinetics with collision efficiencies of 3.3 % and 6.5 %.

In contrast to NO_*x*_, HNO_3_ exhibits reactivity toward both hydrated O**^.^** ^−^ and hydrated OH^−^. Our molecular dynamics simulations reveal that nitric acid is efficiently picked‐up by the water cluster and undergoes ionic dissociation, forming a H_3_O^+^⋅⋅⋅NO_3_^−^ ion pair structure. The proton then almost immediately reacts with O**^.^** ^−^/OH^−^, which thereby leaves the cluster where NO_3_^−^ remains. The mass spectra also demonstrate an efficient HNO_3_ adsorption on the surface of water clusters by observation of (HNO_3_)_2_NO_3_^−^ clusters. However, the overall kinetics of the proton transfer from HNO_3_ to the anions exhibits comparable slow kinetics as measured for NO_*x*_.

## Experimental Section

### Experimental methods

The experiments were performed by using a 4.7 T FT‐ICR mass spectrometer, equipped with a laser vaporization cluster source,^30^ which has recently been modified, providing the capability of generating large hydrated cluster ions, for example, O**^.^** ^−^(H_2_O)_*n*_. The reactant cluster ions are generated in a two‐step process. First, the hydrated electrons (H_2_O)_*n*_ **^.^** ^−^ are formed by laser vaporization of a solid zinc target and jet expansion of the hot plasma in a helium/water gas pulse.^31^ Then, the anionic water clusters are passed through the expansion channel of the source, where they are mixed with N_2_O, yielding hydrated O**^.^** ^−^ and OH^−^ cluster ions.^25^, ^26^

The skimmed cluster beam of O**^.^** ^−^(H_2_O)_*n*_ and OH^−^(H_2_O)_*n*_ ions is transferred through an electrostatic lens system through differential pumping stages into the ultra‐high vacuum (UHV) region of the mass spectrometer, with a background pressure below 4.8×10^−10^ mbar, and is stored in the ICR cell. We avoid kinetic excitation of the ions by not using the so‐called side‐kick, a voltage difference in the entrance electrode of the infinity cell. The trapping potentials are in the range of 1.5 V, posing an upper limit to the kinetic energy of the cluster ions. However, we know from many experiments with ionic water clusters that collisions do not significantly enhance the BIRD rate of water evaporation, which indicates that the available kinetic energy in the center of mass frame of clusters and reactant gas is negligible. Nanocalorimetry yields realistic thermochemical values,^28^ indicating that the kinetic energy of the cluster ions is near‐thermal. Reactant gas (e.g., NO**^.^**, NO_2_ **^.^**, and HNO_3_) is introduced into the UHV region of the mass spectrometer through a leak valve at constant pressures in the range 1.0–8.0×10^−8^ mbar. As the presence of impurities in the samples might interfere with the results, substantial effort has been devoted for their purification. Both gaseous samples, nitric oxide (98.5 %, Sigma–Aldrich) and nitrogen dioxide (≥99.5 %, Sigma–Aldrich) were used directly from the lecture bottle. Before introducing NO**^.^** to the ICR cell, it was flowed through a gas purifier—a glass trap in an ethanol bath at around −50 °C to freeze‐out the residual NO_2_ **^.^**. No such purification method was used for the NO_2_ **^.^** sample. The nitric acid liquid sample (70 %, Sigma–Aldrich) was stored in a glass ampule under vacuum and degassed by several freeze‐pump‐thaw cycles to remove gaseous impurities.

To determine the rate coefficient, reactions are monitored by recording mass spectra as a function of time. The intensities of reactant and product clusters in the mass spectra are summed over all cluster sizes. The kinetic fit yielded a pseudo‐first‐order rate coefficient (*k*_rel_/s^−1^), which is converted to a pressure‐corrected absolute rate coefficient (*k*_abs_/cm^3^ s^−1^). The perfect pseudo‐first‐order behavior also indicates that rate coefficients are largely independent of the cluster size. A relative error of ±30 % is assumed, determined by the uncertainty of the pressure calibration. The absolute rate coefficient *k*_abs_ is then compared with calculated collision rates to determine the reaction efficiency, *Φ*. The reaction efficiency can be estimated by using the hard sphere average dipole orientation (HSA) and the surface charge capture (SCC) models through Equation (11).^32^ As previously shown, the actual collision rate of ionic water clusters lies between the models.^28^, ^33^ If applicable, evaporation of OH**^.^**, which converts O**^.^** ^−^(H_2_O)_*n*_ into OH^−^(H_2_O)_*n*−1_, was included in the fits.

(11) $$\Phi =2k{}_{\mathrm{abs}}/\left(k{}_{\mathrm{HSA}}+k{}_{\mathrm{SCC}}\right)$$

Thermochemistry was investigated by using nanocalorimetry.^28^, 30b The heat released during the reaction is extracted by quantitative modeling of the average size of reactant and product clusters as a function of time, taking into account blackbody infrared radiative dissociation (BIRD).^34^ To extract the reaction enthalpy from the mass spectra, the mean cluster sizes of reactants and products are calculated. The results are fitted with a genetic algorithm by using the following differential equations [Eq. (12), Eq. (13)]:

(12) $$dN{}_{R}=-k{}_{f}\left(N{}_{R}-N{}_{0,R}\right)dt$$

(13) $$dN{}_{P}=-k{}_{f}\left(N{}_{P}-N{}_{0,P}\right)dt+\left(N{}_{R}\Delta N{}_{\mathrm{vap}}-N{}_{P}\right)\left(k\cdot I{}_{R}/I{}_{P}\right)dt$$

Equation (12) and the first term in Equation (13) describe BIRD of water clusters, with *k*_f_ describing the linear dependence of the unimolecular BIRD rate on cluster size. The parameters *N*_0,R_, *N*_0,P_ account for the contribution of the ionic core to the IR absorption cross sections. The second term in Equation (13) describes the evaporation of water molecules owing to the reaction enthalpy released in the water cluster. The average number of evaporated water molecules Δ*N*_vap_ is the key result of the fit. Assuming the energy of 43.3±3.1 kJ mol^−1^ required to evaporate a single water molecule from the water cluster^35^ and minor thermal corrections (see the Supporting Information), this translates to the reaction enthalpy Δ_r_ *H*_exp_(298 K).

### Computational methods

Clusters were calculated at the B3LYP/TZVP level of theory with dispersion correction as suggested by Grimme,^36^ further denoted as B3LYP+D2/TZVP. Structures from our recent article^25^ were used as the initial point for the search of possible conformations. For hydrated NO_3_^−^HNO_3_ and NO_3_^−^(HNO_3_)_2_ clusters with four or more water molecules, a larger conformational space can be expected. Therefore, we ran molecular dynamics at 300 K for 15 ps on the BLYP/SVP potential energy surface with a time step of 30 a.u. (≈0.73 fs). From the last 10 ps, 16 structures were taken and optimized at the B3LYP+D2/TZVP level of theory.

The Gaussian software suite^37^ was used for all quantum chemical calculations included in the present manuscript. For molecular dynamics, the ABIN code was used.^38^

## Conflict of interest

The authors declare no conflict of interest.

## Supporting information

As a service to our authors and readers, this journal provides supporting information supplied by the authors. Such materials are peer reviewed and may be re‐organized for online delivery, but are not copy‐edited or typeset. Technical support issues arising from supporting information (other than missing files) should be addressed to the authors.

Supplementary
