# Supplementary material for: Chemistry of NOx and HNO3 Molecules with Gas‐Phase Hydrated O.− and OH− Ions
Source: Chemistry. 2020 Jun 3;26(35):7861–8. doi: 10.1002/chem.202000322 (PMC7384111; doi:10.1002/chem.202000322)
Supplement: Supplementary file 1 — Supplementary [file CHEM-26-7861-s001.pdf]

Chemistry–A European Journal

Supporting Information

Chemistry of NO_x and HNO_3 Molecules with Gas-Phase Hydrated $O^{\bullet-}$ and OH^- Ions

Jozef Lengyel,*[a, b] Milan Ončák,*[b] and Martin K. Beyer*[b]

S1 Kinetic and nanocalorimetric analysis

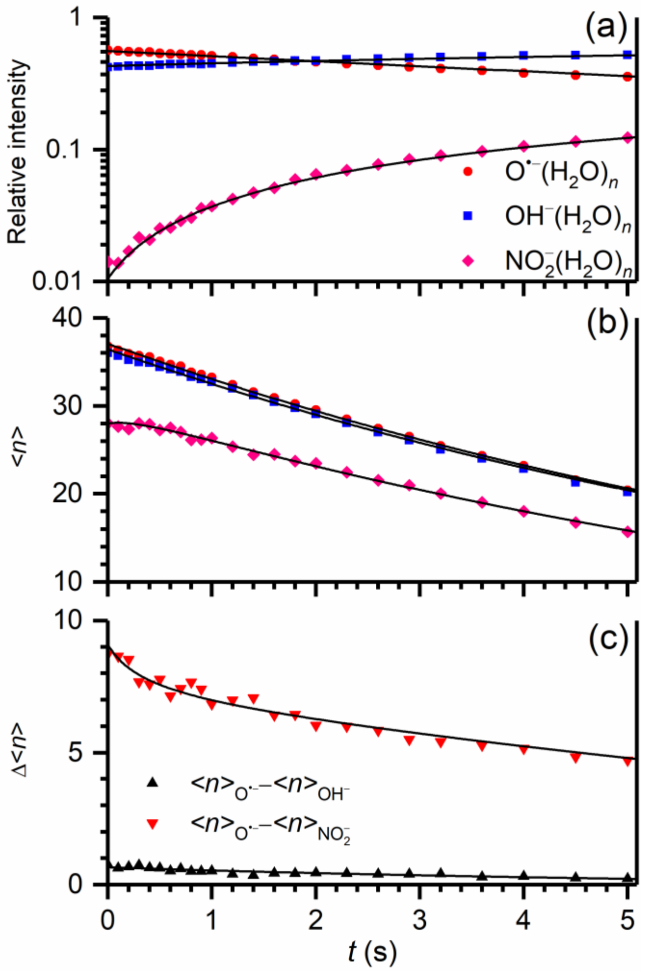

Figure S1. Kinetic (a) and nanocalorimetric (b and c) analysis of the reaction of $NO^{\bullet}$ with $O^{\bullet-}(H_2O)_n$ and $OH^{-}(H_2O)_n$, n = 20-37, ions at a pressure of 4.0×10^{-8} mbar. Panel (a) represents the pseudo-first-order kinetic fit of $O^{\bullet-}(H_2O)_n$ (red circles), $OH^{-}(H_2O)_n$ (blue squares), and $NO_2^{-}(H_2O)_n$ (magenta diamonds). Panel (b) shows the fit of the cluster men sizes for the reactant and product ions, and panel (c) illustrates the fit of their size difference for reaction R2 [$\Delta(<n>O^{\bullet-}-<n>OH^{-})$; black triangle up] and reaction (R3) [$\Delta(<n>O^{\bullet-}-<n>NO_2^{-})$; red triangle down].

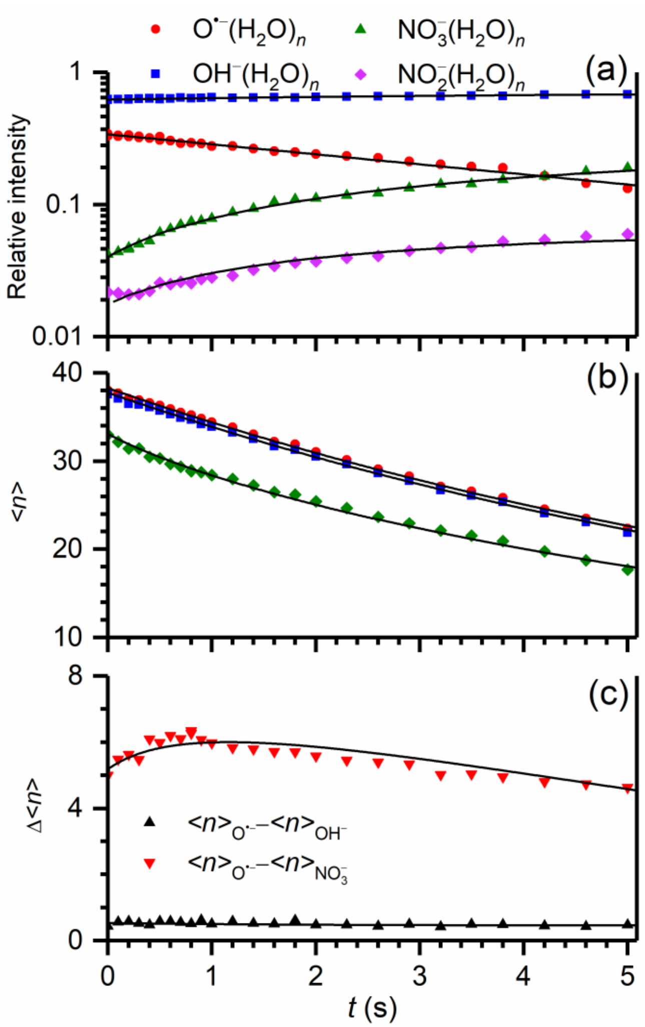

Figure S2. Kinetic (a) and nanocalorimetric (b and c) analysis of the reaction of $NO_2^{\bullet}$ with $O^{\bullet-}(H_2O)_n$ and $OH^-(H_2O)_n$, n = 22–38, ions at a pressure of 6.3×10^{-8} mbar. Panel (a) represents the pseudo-first-order kinetic fit of $O^{\bullet-}(H_2O)_n$ (red circles), $OH^-(H_2O)_n$ (blue squares), $NO_3^-(H_2O)_n$ (green triangles), and $NO_2^-(H_2O)_n$ (pink diamonds). Panel (b) shows the fit of the cluster mean sizes for the reactant and product ions, and panel (c) illustrates the fit of their size difference for reaction R2 [$\Delta(\langle n\rangle O^{\bullet-}-\langle n\rangle OH^-)$; black triangle up] and reaction R5 [$\Delta(\langle n\rangle O^{\bullet-}-\langle n\rangle NO_3^-)$; red triangle down].

S2 Thermochemistry

S2.1 Conversion of ΔE_{raw} to $\Delta_r H_{exp}$(298K)

Expected temperatures in the experiment for reaction:

$(H_2O)_nO^{\bullet-}$	+	$NO^{\bullet}$	$\longrightarrow$	$(H_2O)_mNO_2^-$	+ $(n-m)$ H_2O
90 - 120 K		298 K		90-120 K	

The internal temperature of $(H_2O)_nO^{\bullet-}$ is a result of radiative heating and evaporative cooling. We assume a value of 90–120 K, which corresponds to the region where the solid-to-liquid phase transition of the anionic water cluster occurs.[1] The neutral reactant is at room temperature, equilibrated in collisions with the surfaces in the UHV region of the mass spectrometer. The neutral products, including the evaporating H_2O molecules, will have an internal energy distribution that corresponds to the internal temperature of the cluster after the reaction. The reaction enthalpy is heating the cluster with 265 kJ mol^{-1}. Setting n = 30, there are about 6n = 180 low-lying vibrational degrees of freedom which correspond to the translational and rotational degrees of freedom of the free water molecules. If we assume that these are thermally populated, we have 1.47 kJ mol^{-1} per degree of freedom. A fully populated vibrational degree of freedom contains RT internal energy, therefore the 1.47 kJ mol^{-1} per degree of freedom correspond to a temperature increase of 177 K. Of course, the cluster immediately responds with evaporative cooling, therefore we do not know the exact temperature at which each neutral molecule evaporates. In addition, instead of increasing the temperature, the cluster may convert the additional energy into latent heat by breaking hydrogen bonds. Since a detailed modeling of all these aspects goes beyond the scope of the present work, we give the conservative range above.

From nanocalorimetry, we obtain ΔE_{raw} at these conditions:

$\Delta E_{raw} = -\Delta_{vap}N\ \Delta_{vap}E = -265 \pm 47$ kJ mol^{-1}

Corrections for $\Delta_r H_{exp}$(298K):

The difference in heat capacity of $(H_2O)_nO^{\bullet-}$ compared to $(H_2O)_mNO_2^-$ is unknown. However, the three translational degrees of freedom of $O^{\bullet-}$ ion oscillating in the cluster are converted to six low-lying degrees of freedom of the NO_2^- ion oscillating in the cluster provide an upper limit. If these low-lying modes are thermally populated, we have a contribution to the heat capacity of 3R. Since the heat of the reaction has to provide the energy to populate these modes, this effect reduces the exothermicity, therefore the correction has a positive sign. Since the correction lies somewhere between 0 and 3 RT, we suggest:

$\Delta(\Delta H)C = (1.5 \pm 1.5)\ [R(298\text{ K} - 105\text{ K})] = 2.4 \pm 2.4$ kJ mol^{-1}

In summary, we obtain with Gaussian error propagation:

$\Delta_r H_{exp}(298K) = \Delta E_{raw} + \Delta(\Delta H)C = -265+2.4 \pm \text{sqrt}(47+2.4)$ kJ mol^{-1} $= -263 \pm 47$ kJ mol^{-1}

S2.2 Thermochemical Cycles

(R5) $O^{\bullet-}(aq) + NO_2^{\bullet}(g) \rightarrow NO_3^{-}(aq)$

$\Delta_r H_{exp}(R5) = \Delta H_f(NO_3^{-}(aq)) - \Delta H_f(NO_2^{\bullet}(g)) - \Delta H_f(O^{\bullet-}(aq))$

$\Delta_r H_{exp}(R5) = –286±42$	this work
$\Delta H_f(NO_3^{-}(aq))$ = –205 kJ mol^{-1}	[2]
$\Delta H_f(NO_2^{\bullet}(g))$ = 33.18 kJ mol^{-1}	[2]

yields $\Delta H_f(O^{\bullet-}(aq))$ = 48±42 kJ mol^{-1}

(R3) $O^{\bullet-}(aq) + NO^{\bullet}(g) \rightarrow NO_2^{-}(aq)$

$\Delta H_f(O^{\bullet-}(aq))$ = 48±42 kJ mol^{-1}	this work
$\Delta H_f(NO^{\bullet}(g))$ = 90.25 kJ mol^{-1}	[2]
$\Delta_r H_{exp}(R5)$ = –263±47 kJ mol^{-1}	this work

yields $\Delta H_f(NO_2^{-}(aq))$ = –125±63 kJ mol^{-1}

(R7+R7") $O^{\bullet-}(aq) + HNO_3(g) \rightarrow NO_3^{-}(aq) + OH^{\bullet}(g)$

$\Delta H_f(O^{\bullet-}(aq))$ = 48 ±42 kJ mol^{-1}	this work
$\Delta H_f(HNO_3(g))$ = –134.3 kJ mol^{-1}	[2]
$\Delta H_f(NO_3^{-}(aq))$ = –205 kJ mol^{-1}	[2]
$\Delta H_f(OH^{\bullet}(g))$ = 39 kJ mol^{-1}	[3]

yields $\Delta_r H_{exp}$(R7+R7") = –80±42 kJ mol^{-1}

(R8) $OH^{-}(aq) + HNO_3(g) \rightarrow NO_3^{-}(aq) + H_2O(g)$

$\Delta H_f(OH^{-}(aq))$ = –230 kJ mol^{-1}	[2]
$\Delta H_f(HNO_3(g))$ = –134.3 kJ mol^{-1}	[2]
$\Delta H_f(NO_3^{-}(aq))$ = –205 kJ mol^{-1}	[2]
$\Delta H_f(H_2O(g))$ = –241.8 kJ mol^{-1}	[2]

yields $\Delta_r H_{exp}$(R8) = –82.5 kJ mol^{-1}

S3 Selected structures of hydrated reactant and product ions

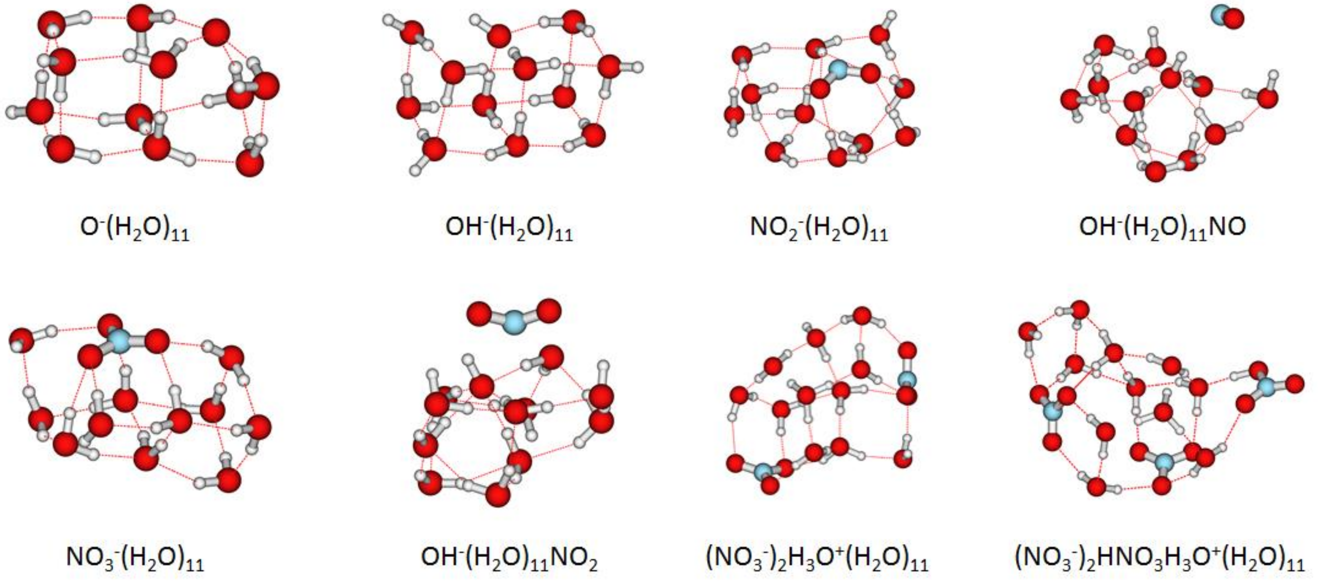

Figure S3. Calculated structures with 11 water molecules optimized at the B3LYP+D2/TZVP level of theory.

References

[1] C. Hock, M. Schmidt, R. Kuhnen, C. Bartels, L. Ma, H. Haberland, B. von Issendorff, *Phys Rev Lett* **2009**, *103*, 073401.

[2] Atkins, P. and de Paula, J.: Atkins' Physical Chemistry, 8th ed., W. H. Freeman and Co., New York, 2006.

[3] NIST Chemistry WebBook, NIST Standard Reference Database Number 69, National Institute of Standards and Technology, https://doi.org/10.18434/T4D303.

Cartesian coordinates (in Å) of the most stable structures found along with enthalpies as calculated at the B3LYP+D2/TZVP level (in a.u.)

H2O
H=-76.435717
O 0.000000 0.117093 0.000000
H 0.764268 -0.468556 0.000000
H -0.764268 -0.468188 0.000000

(H2O)2
H=-152.879567
H -1.897093 0.024870 0.748304
H -0.530713 0.004454 0.045254
O -1.487826 -0.003960 -0.121446
H 1.710591 0.754892 -0.382013
H 1.700292 -0.780474 -0.336448
O 1.364941 0.003492 0.112059

(H2O)4
H=-229.335115
O -0.113539 1.582766 0.111392
H 0.672590 1.000023 0.077378
H -0.019002 2.205452 -0.615774
O 1.435299 -0.685648 -0.083746
H 1.951600 -1.134743 0.591835
H 0.546647 -1.095053 -0.076700
O -1.322419 -0.888170 -0.095174
H -1.944138 -1.129126 0.597795
H -1.202426 0.081862 -0.034313

(H2O)4
H=-305.792958
O 0.405501 -1.864333 0.011141
H -0.491048 -1.448945 -0.020134
H 0.404727 -2.453594 0.771203
H 1.450089 -0.491434 0.019923
O 1.864854 0.405394 -0.011321
H 2.454638 0.404691 -0.770972
O -1.864859 -0.405455 -0.011132
H -2.454071 -0.405320 -0.771230
H -1.450399 0.491517 0.019993
O -0.405545 1.864442 0.011309
H -0.404523 2.453650 0.771411
H 0.490984 1.449049 -0.020162

(H2O)5
H=-382.244397
O 2.049551 -0.998915 -0.035750
O -0.321761 -2.248451 -0.094151
O -2.237933 -0.396995 0.176271
O -1.077244 1.995960 -0.136996
O 1.574835 1.651361 0.029868
H 2.583124 -1.458165 0.618786
H -0.544959 -2.736006 -0.892541
H -2.754418 -0.351115 0.985954
H -1.232882 2.467673 -0.960472
H 2.047506 2.036631 0.772951
H 1.187435 -1.485927 -0.078573
H -1.055564 -1.596212 0.041144
H -1.847865 0.504516 0.043260
H -0.094107 1.904426 -0.054626
H 1.812144 0.690500 0.010180

(H2O)6
H=-458.699747
O 1.488292 -0.139162 1.418233
H 0.558010 -0.485699 1.576042
H 2.012122 -0.329876 2.201483
O 0.676710 1.943016 -0.296785
H 1.047431 1.515783 0.496069
H 1.059211 1.414028 -1.014095
O 1.651996 -0.561547 -1.266010
H 2.402401 -1.000873 -1.677084
H 1.795243 -0.577087 -0.294000
O -1.889783 1.119337 -0.186333
H -1.001539 1.551129 -0.251953
H -2.550775 1.802836 -0.329616
O -1.063043 -1.453322 -1.194359
H -0.139287 -1.236732 -1.411048
H -1.521555 -0.596776 -1.184693
O -1.000154 -0.963121 1.515133
H -1.048324 -1.447781 0.661140
H -1.525083 -0.170570 1.308726

(H2O)7
H=-535.156231
O -0.929767 -0.713395 -1.664182
O -2.804084 0.623365 -0.217902
O -1.078195 0.143853 1.785959
O 0.167035 -2.083068 0.548121
H -0.672945 -1.381198 -1.001803
H -1.719337 -0.265142 -1.282648
H -3.706803 0.361603 -0.016303
H -2.277278 0.505532 0.613011
H -0.345766 0.769859 1.631691
H -0.716776 -0.725322 1.534316
H 0.276631 -3.005368 0.797587
H 1.073656 -1.684631 0.469538
O 1.088231 1.756459 0.951420
O 2.425819 -0.682125 0.234494
O 1.103931 0.906625 -1.630876

H 2.213656 -0.226932 -0.603238
H 2.360994 0.034730 0.882958
H 1.132899 2.694610 1.158648
H 1.034949 1.673967 -0.027530
H 0.288104 0.318225 -1.728855
H 1.274249 1.316355 -2.483645

(H2O)8
H=-611.620651
O 1.441691 1.963227 -0.085612
H 2.067306 2.667545 -0.279113
H 1.497718 1.291324 -0.826746
O -1.442322 0.087228 1.962727
H -2.068145 0.281343 2.666691
H -1.497254 0.828415 1.290792
O -1.439375 -0.086168 -1.964814
H -1.495905 -0.827289 -1.292939
H -2.064315 -0.279812 -2.669692
O 1.345684 -0.031224 1.872417
H 0.403630 0.014194 2.128232
H 1.504853 0.778591 1.349365
O -1.346406 1.871955 0.029729
H -1.504557 1.348748 -0.780185
H -0.404342 2.127931 -0.014666
O 1.348566 0.030264 -1.870479
H 0.406816 -0.014479 -2.127551
H 1.506435 -0.779644 -1.347179
O 1.440042 -1.964320 0.087678
H 2.065051 -2.668927 0.282092
H 1.495389 -1.292448 0.828895
O -1.347861 -1.870962 -0.031664
H -0.406100 -2.127801 0.014090
H -1.506740 -1.347685 0.778062

(H2O)9
H=-688.073438
O 0.395300 -1.147649 2.076270
O 0.343540 1.462649 1.729985
O -1.657286 1.980322 -0.141053
O -1.418265 -2.003734 0.133584
O -3.242602 -0.108342 -0.067964
O 2.366820 -1.262697 0.103072
O 2.453839 1.362683 -0.088620
O 0.345669 1.135240 -1.902031
O 0.431113 -1.496598 -1.890513
H 1.817658 -1.530046 -0.660012
H 1.794112 -1.400551 0.882849
H -0.834166 -1.967742 -0.649388
H -0.834934 -1.810246 0.893393
H -0.408017 1.485576 -1.386775
H 1.143421 1.386261 -1.397832
H 1.143521 1.620095 1.191806
H -0.406621 1.743191 1.169220
H 0.329693 -0.148379 2.031904
H 0.427187 -1.396904 3.004466
H 0.350129 -0.502291 -1.985873
H 0.450287 -1.873056 -2.775209
H 2.550965 0.367689 -0.015970
H 3.335216 1.745176 -0.123637
H -2.333124 1.243816 -0.090541
H -2.136144 2.799659 -0.297037
H -3.929364 -0.260762 0.587048
H -2.604838 -0.876483 -0.000245

(H2O)10
H=-764.530464
O -0.653698 -2.330443 1.312353
H -1.197228 -1.510949 1.538580
H -0.947297 -3.043479 1.886940
O -2.045861 -0.159151 1.658065
H -2.436144 0.011806 0.783507
H -1.468458 0.626005 1.827775
O -0.980880 -1.890042 -1.408864
H -0.067688 -1.687706 -1.700682
H -0.890286 -2.252783 -0.504541
O 1.959518 -1.529310 0.897131
H 1.083201 -1.881930 1.157018
H 1.979540 -1.583587 -0.076966
O -0.398128 1.995587 1.738007
H -0.559035 2.339746 0.842966
H 0.536722 1.713102 1.736084
O 1.637614 -1.150642 -1.867349
H 1.676615 -0.152932 -1.764144
H 2.200236 -1.389919 -2.609608
O 2.203403 1.045006 1.348666
H 2.971008 1.249691 1.890562
H 2.144958 0.046527 1.269937
O -0.906194 2.381771 -1.043569
H -1.549672 1.639101 -1.161096
H -1.268226 3.152604 -1.490546
O -2.491116 0.209637 -1.140213
H -1.918483 -0.604153 -1.314353
H -3.301046 0.101560 -1.647617
O 1.699625 1.420860 -1.355083
H 2.006181 1.455128 -0.426517
H 0.810840 1.825978 -1.350451

(H2O)11
H=-840.982286
O -0.870128 -1.690967 0.279140
H -1.817755 -1.954350 0.257949
H -0.756359 -1.051537 -0.455865
O 2.771215 1.206322 -0.307713
H 2.708444 0.528733 -1.009386
H 2.029451 1.819891 -0.460622
O -3.572629 -1.766074 0.108053

H -3.534613 -0.857927 -0.277113
H -4.158726 -2.282815 -0.451951
O -0.318173 0.105162 -1.789229
H -0.037366 1.005225 -1.532807
H 0.494600 -0.337901 -2.104872
O -2.980082 0.669713 -0.932922
H -2.227266 0.441377 -1.501977
H -2.591983 1.215826 -0.224030
O -1.489811 2.110062 1.045277
H -1.920835 2.700196 1.670946
H -1.046031 1.370471 1.583299
O -0.363039 0.130834 2.247211
H 0.613234 0.108450 2.289278
H -0.605288 -0.630242 1.670721
O 0.378837 2.610799 -0.775626
H -0.305743 2.592106 -0.057371
H 0.253226 3.421100 -1.278469
O 2.384928 -0.171100 1.907473
H 3.092307 -0.065887 2.550576
H 2.599601 0.410107 1.122339
O 1.789472 -2.366675 0.248686
H 2.079687 -1.768758 0.963852
H 0.814093 -2.396538 0.327587
O 2.204606 -0.990133 -1.945547
H 2.735379 -1.446974 -2.604627
H 2.100371 -1.604097 -1.155876

HO-.NO
H=-205.777456
O -1.314566 -0.250391 0.000075
N 0.365397 0.552865 0.000099
O 1.219136 -0.306840 -0.000074
H -1.794334 0.587799 -0.000703

HO-.NO(H2O)
H=-282.250005
O 0.049803 1.353137 -0.225168
N -1.343079 0.046597 0.315623
O -1.110258 -1.004415 -0.205261
H -0.030547 1.883095 0.576801
H 1.263032 -1.216190 0.049973
O 1.917710 -0.508764 0.080818
H 1.311032 0.287250 -0.039252

HO-.NO(H2O)2
H=-358.716715
O -0.169487 -0.902651 0.419192
N -0.166021 1.208451 0.481485
O 0.669316 1.609055 -0.250911
O -2.684307 -0.510233 -0.376180
H -0.224310 -1.011583 1.375430
H -2.577856 0.445755 -0.327547
H -1.759296 -0.800178 -0.119185
H 2.450875 -0.002521 -0.439799
O 2.412586 -0.950155 -0.271425
H 1.447872 -1.058759 -0.024700

HO-.NO(H2O)3
H=-435.177569
O 1.950199 -1.500912 0.000673
O -1.934475 -1.134959 -0.600971
N -1.246373 -1.397371 0.309801
O 0.123150 0.315769 0.666601
O -1.961287 1.896522 -0.051489
H 0.055165 0.314335 1.627810
H 1.416396 -2.095466 -0.534795
H 1.268744 -0.831395 0.315522
H -1.132631 1.408236 0.206467
H -2.484136 1.196548 -0.455127
O 2.479831 1.424053 -0.367662
H 2.756146 0.510077 -0.517997
H 1.585591 1.275468 0.012293

HO-.NO(H2O)4
H=-511.644028
O 0.379559 -0.034385 -1.185171
O -0.340514 -2.103138 0.234058
O -0.424661 2.123947 0.038796
O -2.363911 -0.080148 -0.986701
O -1.399376 0.065516 1.779435
H 0.785639 -0.065712 -2.054438
H 0.094630 -1.400429 -0.343343
H -1.226161 -2.137648 -0.147957
H 0.036899 1.385118 -0.469777
H -1.310488 2.088938 -0.342825
H -1.403011 -0.073918 -1.226017
H -2.331965 -0.034272 -0.015661
H -0.902842 -0.712728 1.457705
H -0.933726 0.830868 1.388302
N 2.663335 -0.180067 -0.402303
O 2.717363 0.200739 0.690850

HO-.NO(H2O)5
H=-588.104886
O -0.889710 -0.849868 -1.270260
O 0.266019 1.487745 -0.929748
O -3.336033 -0.074928 -0.549247
O 1.398076 -1.314952 0.094341
O -1.297110 -1.194002 1.439576
O -1.709908 1.642243 1.058730
H -0.922316 -1.366965 -2.079004
H -1.199226 -1.292755 0.466067
H -1.365400 -0.224764 1.540207
H -2.421115 1.290828 0.489817
H -0.962938 1.746680 0.430437
H -0.197599 0.646825 -1.224357

H 1.063144 1.145696 -0.505964
H 0.926529 -1.355084 0.938725
H 0.635959 -1.191578 -0.537239
H -2.454109 -0.335426 -0.954262
H -3.364979 -0.647724 0.227315
O 4.090503 0.068146 -0.078227
N 3.155337 0.495599 0.440991

HO-.NO(H2O)6
H=-664.565638
O -0.106098 -0.450999 -1.393919
O 1.328977 -0.850824 0.656600
O 0.503485 1.914462 -0.285609
O -2.063060 -2.009912 -0.354430
O -1.184717 -0.785645 2.011200
O -2.756037 0.563018 -1.370174
O -1.976917 1.765126 1.079633
H -1.825639 0.310134 -1.550491
H -2.678588 1.069258 -0.536058
H -1.266257 -1.619536 -0.798637
H -2.726415 -1.346516 -0.613841
H 0.792938 -0.762449 -0.230488
H 1.564422 -1.779856 0.735413
H -1.126576 1.995045 0.654169
H -1.781604 0.913891 1.525271
H -0.255431 -0.731023 1.718236
H -1.600585 -1.309737 1.291766
H 0.250140 1.122959 -0.838210
H 1.120506 1.529160 0.347402
H 0.293246 -0.630104 -2.249368
O 4.094964 0.509523 -0.030873
N 3.787868 -0.571317 -0.279227

HO-.NO(H2O)7
H=-741.026174
O 0.095372 -2.457234 0.143105
O 1.849779 -0.271651 0.451549
O 0.127451 1.068880 -1.012491
O -1.113254 -0.951831 -1.896441
O -0.512578 1.497970 1.572585
O -1.666286 -1.083860 1.868640
O -3.385355 -0.521739 -0.263400
O -2.504067 2.148481 -0.428812
H -0.596096 -0.093954 -1.626873
H -1.018541 -1.053387 -2.846615
H -0.342124 -2.101990 -0.655555
H 0.842067 -1.840447 0.279914
H 1.273088 0.248387 -0.194722
H 1.600162 0.095732 1.308146
H -1.066693 -1.676759 1.368990
H -1.214572 -0.215060 1.866378
H -0.176042 1.419852 0.643410
H -1.363264 1.933734 1.403635
H -1.636350 1.866461 -0.778723
H -2.989348 1.300013 -0.355787
H -2.922966 -0.765034 0.569128
H -2.735162 -0.760293 -0.950077
H 0.546989 1.712609 -1.589014
O 4.852644 -0.104518 -0.350720
N 4.264172 0.762022 0.126522

HO-.NO(H2O)8
H=-817.483831
O -0.656895 0.666337 -1.819418
O -1.761461 -1.405771 -0.715617
O -1.326328 -1.057131 1.797351
O 0.730430 -2.428195 -0.863592
O 1.866411 -0.479745 -2.338728
O -1.812945 2.719721 -0.318921
O 0.319238 1.000556 0.776602
O -3.430399 0.496719 0.579525
O 1.372051 -1.711136 1.859916
H -1.153643 -0.164172 -1.530314
H 0.162839 0.355468 -2.249715
H 2.331004 0.032823 -1.667376
H 1.518463 -1.266706 -1.837721
H -0.217249 -2.116992 -0.835850
H 1.035630 -2.363731 0.059747
H -2.974321 -0.150078 -0.000999
H -3.050652 1.353456 0.311986
H -1.139110 2.529997 0.351696
H -1.495023 2.177302 -1.067157
H 0.089591 0.870521 -0.171190
H -0.249853 0.343106 1.230466
H 0.427349 -1.606006 2.084368
H 1.621122 -0.822072 1.573057
H -1.511744 -1.350725 0.842000
H -2.099286 -0.500195 1.978169
H -2.362155 -2.041672 -1.111620
N 2.939889 0.932678 0.497144
O 3.260876 1.972512 0.862937

HO-.NO(H2O)9
H=-893.943803
O 0.153230 1.110235 -1.844430
O -1.165074 -1.116799 -1.514213
O -1.160297 -1.531289 1.045176
O 1.381335 -2.098560 -1.343687
O 2.777116 0.114198 -1.966221
O 1.440735 -2.158397 1.512281
O 0.329342 0.824078 0.948322
N 2.915105 0.809847 1.313054
O 3.087743 1.729718 1.978499
O -1.457221 2.778506 -0.345805
O -3.180783 0.548661 -0.738639
H -0.365975 0.248972 -1.885717

H 1.070842 0.897698 -2.103888
H 3.019520 0.438154 -1.091106
H 2.348198 -0.765563 -1.782760
H 0.440716 -1.791566 -1.431965
H 1.494666 -2.280903 -0.391404
H -2.475954 -0.062012 -1.084675
H -2.729891 1.410403 -0.642538
H -1.021046 2.401196 0.434475
H -0.886628 2.436097 -1.063462
H 0.382979 0.895787 -0.029684
H -0.229542 0.027015 1.088336
H 0.460188 -2.107694 1.505274
H 1.703551 -1.232154 1.589002
H -1.244498 -1.499382 0.037842
H -2.045435 -1.326419 1.404366
H -1.566371 -1.671768 -2.188041
O -3.668430 -0.441493 1.691920
H -3.590502 -0.049335 0.775640
H -3.472119 0.291692 2.283300

HO-.NO(H2O)10
H=-970.401047
O 2.314573 0.375370 -1.142310
O 2.098605 -2.280425 -1.390474
O 0.901207 0.612495 1.178999
O 0.245139 2.870748 0.197839
O -0.177140 -1.494547 0.058063
O -2.469396 -2.040960 1.475306
O -1.872288 0.127555 -1.416773
O -2.428266 2.103207 0.371631
O -3.880388 -1.687665 -1.013759
O -1.617248 0.494325 2.433068
O 0.262199 1.776448 -2.390332
H 1.856733 0.491928 -0.268671
H 1.711779 0.835541 -1.766672
H -0.509601 1.187108 -2.282103
H 0.239523 2.329912 -1.585006
H 0.555862 2.005617 0.666532
H 0.793385 3.586851 0.527445
H -1.616433 -2.060669 0.995111
H -3.123512 -1.912205 0.763932
H -3.545058 -2.459680 -1.480352
H -3.251229 -0.965734 -1.260368
H 0.518906 -1.943527 -0.460438
H 0.287505 -0.756549 0.557844
H -0.706397 0.496518 2.060419
H -1.983024 -0.390851 2.229210
H 2.235363 -1.293642 -1.422654
H 2.786215 -2.586692 -0.790838
H -1.235418 -0.438259 -0.921657
H -2.130018 0.855123 -0.791824
H -1.595010 2.607235 0.315195
H -2.309221 1.579402 1.199388
H 1.510891 0.547536 1.918940
O 4.053060 -0.225770 1.386261
N 4.295473 -0.965885 0.545203

HO-.NO(H2O)11
H=-1046.855352
O -0.217293 -2.649666 0.133019
O 0.012508 -0.212810 -0.992805
O -1.915840 0.138144 0.776874
O -3.037134 -2.259224 0.148404
O -1.904873 2.822064 1.304258
O 0.782783 2.868049 1.345342
O 1.117557 2.347806 -1.299815
O -0.255092 -1.239217 2.565196
O 1.700338 0.115039 1.235279
O 3.623984 1.194750 -0.810458
O 2.533141 -1.091849 -1.861511
O 2.576046 -2.611179 0.516643
H -0.121128 -1.785979 -0.357719
H -1.175330 -2.825079 0.116354
H -3.375444 -2.112076 -0.740266
H -2.794551 -1.360760 0.471034
H -1.169514 0.093901 0.102259
H -2.040155 1.087296 1.006607
H 1.618531 -0.805751 -1.639336
H 2.712491 -1.782919 -1.195110
H 2.494077 -1.734885 0.930663
H 1.654901 -2.917522 0.444693
H 1.084895 -0.313789 1.878360
H 1.207411 0.081575 0.392272
H -0.917787 2.881028 1.424507
H -2.071124 3.266722 0.466779
H -0.972507 -0.689029 2.192003
H -0.195214 -1.950353 1.895987
H 0.669868 1.472198 -1.248870
H 2.068890 2.111326 -1.284611
H 3.385802 0.355023 -1.270911
H 3.387557 1.013891 0.109477
H 0.923933 2.858566 0.366242
H 1.154766 2.005866 1.601784
H -0.411707 -0.239507 -1.856410
N -3.266618 0.889180 -1.542920
O -3.047668 -0.038659 -2.186094

HNO3-
H=-281.006470
O -0.714928 1.138621 -0.060394
N -0.349507 -0.023247 0.168568
O 1.873796 -0.221822 -0.036994
O -1.073719 -0.988047 -0.058523
H 1.765358 0.732706 0.067316

HNO3-.(H2O)

H=-357.474359
O -2.356469 -0.436354 -0.218704
O 0.527571 -1.245645 0.335146
N 0.869458 -0.166173 -0.139584
O -0.511031 1.484488 0.307992
O 2.035943 0.196928 -0.227488
H -0.159854 1.909445 -0.484689
H -1.689268 -1.115218 -0.055522
H -1.805198 0.373656 -0.058263

HNO3-.(H2O)2
H=-433.933680
N 1.312346 0.312996 -0.091875
O 1.215141 0.051833 -1.280433
O 0.939268 1.370419 0.417587
O 0.707905 -1.326095 1.118875
H 0.552772 -0.712327 1.848280
O -2.115881 1.229369 0.206511
H -2.017702 0.280294 -0.015070
H -1.197459 1.526966 0.261815
H -0.633918 -1.454272 0.180676
O -1.372020 -1.412305 -0.505217
H -0.885422 -1.137403 -1.291160

HNO3-.(H2O)3
H=-510.390299
O 2.367750 -0.170594 -1.060320
O 0.517731 -1.375849 1.197307
N -0.946412 -1.090336 -0.194160
O -0.508590 -0.512591 -1.213705
O -1.985578 -0.746982 0.345259
O 1.236768 1.272891 1.160282
H 1.207214 -1.583304 0.547076
H 1.464490 -0.401969 -1.329072
H 2.202586 0.446611 -0.321677
H 0.479233 1.717867 0.746917
H 0.914767 0.350216 1.324059
H -1.832928 1.542405 0.263242
O -1.236306 2.057185 -0.297391
H -0.944673 1.368049 -0.922872

HNO3-.(H2O)4
H=-586.857990
O 0.215092 -0.061339 -1.057457
N 2.237261 0.168314 0.108613
O 2.117290 -0.200041 1.244309
O -2.024898 0.034668 1.614987
O -0.937628 -2.143715 0.092693
O -0.633748 2.108940 0.196964
O -2.548523 0.144619 -1.238047
H 0.731478 -0.058670 -1.868233
H -0.343349 -1.470256 -0.346863
H -1.750411 -2.043566 -0.418394
H -0.151617 1.376615 -0.284274
H -1.454038 2.162358 -0.308926
H -1.570131 0.081371 -1.342622
H -2.652568 0.127127 -0.269771
H -1.540031 -0.768475 1.343175
H -1.429524 0.772613 1.381009
O 3.124835 -0.052796 -0.684123

HNO3-.(H2O)5
H=-663.315664
O -0.975829 -1.152218 -0.859553
O 0.062687 1.266071 -0.889317
O 3.568159 0.866653 0.638484
N 3.044010 0.202184 -0.205515
O 0.964737 -0.929138 1.017939
O -3.529698 -0.427375 -0.947808
O -1.963233 -0.699622 1.682781
O -2.397701 1.838706 0.355395
H -0.802626 -1.879000 -1.463067
H -1.653682 -1.075064 0.828512
H -2.105620 0.239564 1.456937
H -2.931503 1.255180 -0.218182
H -1.517001 1.824016 -0.075675
H -0.326094 0.360696 -1.084110
H 0.629093 1.056486 -0.135241
H 0.325109 -0.753507 1.722555
H 0.355068 -1.134237 0.256964
H -2.571926 -0.718890 -1.051813
H -3.735342 -0.754379 -0.063342
O 3.399185 -0.742597 -0.839787

HNO3-.(H2O)6
H=-739.777293
O -0.941699 -0.348830 -1.626537
O 1.061104 -1.085484 -0.262037
O 3.133668 1.120326 -0.170656
N 3.329953 0.040306 0.298275
O -0.017859 1.966542 -0.665385
O -2.493124 -1.998779 -0.129005
O -0.819686 -0.975944 1.883830
O -3.409749 0.651180 -0.607571
O -1.799755 1.652086 1.498961
H -2.604372 0.426590 -1.118647
H -3.038096 1.088213 0.185961
H -1.879559 -1.565656 -0.776365
H -3.184705 -1.314688 -0.088822
H 0.276001 -0.818065 -0.889767
H 1.115146 -2.044937 -0.305014
H -1.154337 1.921486 0.813911
H -1.481081 0.767067 1.774533
H -0.049051 -0.924350 1.287035
H -1.482748 -1.433420 1.322633
H -0.399157 1.157385 -1.112444

H 0.861016 1.684768 -0.389034
H -0.874819 -0.440697 -2.580865
O 4.110363 -0.829327 0.052019

HNO3-.(H2O)7
H=-816.238457
O -2.431431 -0.084872 2.024666
O -2.886724 1.830975 0.029324
O -0.313033 1.499568 -0.553526
O 2.872248 1.443985 0.200419
N 3.227798 0.336455 -0.086387
O 0.335377 0.316390 1.600691
O -1.695007 -0.082470 -2.193975
O -2.288906 -1.921593 -0.101562
O 0.447221 -2.214107 0.334841
O 1.273102 -0.511263 -1.711665
H 0.124120 0.857327 0.745179
H 1.030602 0.795567 2.059163
H -1.466165 0.042795 2.090716
H -2.720579 0.653376 1.448608
H -1.902221 1.835594 -0.155694
H -3.238712 1.301450 -0.695656
H -2.444471 -1.365256 0.691571
H -2.200096 -1.287639 -0.841446
H -1.208394 0.605193 -1.663530
H -0.960065 -0.587816 -2.566890
H 0.786551 0.251397 -1.327116
H 1.065490 -1.242579 -1.088543
H -0.520500 -2.276591 0.172405
H 0.521346 -1.452993 0.940176
H 0.149084 2.307030 -0.793311
O 3.485833 -0.625617 0.569422

HNO3-.(H2O)8
H=-892.695582
O -2.062624 -0.838460 1.903475
O -1.837675 1.849969 1.531856
O -2.411341 1.400300 -0.973518
O 0.884264 1.495129 1.395162
N 2.982233 -0.724302 0.320594
O 3.488662 -0.138335 -0.597493
O 0.324749 -1.061568 0.561629
O 1.807950 2.362700 -1.203206
O -1.107466 -2.376856 -1.350593
O 0.067870 0.342918 -1.743173
O -3.506221 -1.207846 -0.519350
H -1.176330 -0.968862 1.481068
H -2.695899 -1.067130 1.194266
H -2.759457 -1.730917 -0.882006
H -3.293899 -0.292575 -0.772988
H -2.751950 2.194038 -1.394756
H -2.208801 1.646991 -0.011552
H -2.024792 0.930339 1.846702
H -0.852291 1.892739 1.562624
H -0.822973 -1.775343 -2.051389
H -0.589085 -2.031997 -0.575801
H -0.815250 0.718613 -1.548453
H 0.289148 -0.187881 -0.939512
H 0.743077 0.545032 1.152493
H 0.858104 -1.717285 1.018571
H 2.638347 1.874871 -1.181053
H 1.164104 1.691774 -1.539225
H 1.221475 1.916911 0.583728
O 3.376937 -1.649101 0.971852

HNO3-.(H2O)9
H=-969.155142
O 3.129458 -1.394126 -0.344066
O 2.621172 1.126190 -0.389798
O 0.908455 3.143567 -1.075214
O -0.675443 2.994394 1.188233
O -4.093101 -0.402299 0.938600
N -3.814285 -0.137472 -0.191232
O -1.155021 0.426443 0.161923
O 1.187559 0.831693 1.836830
O 0.528547 0.367780 -1.995626
O 1.851168 -1.885378 2.105306
O -0.535475 -2.319015 0.679247
O 0.868697 -2.356155 -1.728876
H 1.742585 -2.182786 -1.330922
H 0.284663 -2.471352 -0.945883
H 1.385008 0.598491 -1.569341
H 0.563932 -0.610693 -2.102355
H 0.436991 2.661943 -1.766764
H 1.580998 2.475936 -0.776089
H 2.963707 -0.376375 -0.371711
H 3.454114 1.596638 -0.487955
H -0.618158 0.469248 -0.668475
H -1.246429 1.349279 0.480168
H -0.887087 -1.412672 0.648919
H 0.240631 -2.268178 1.278213
H 1.828799 1.019703 1.094286
H 2.410876 -1.862997 1.304912
H 1.693851 -0.934778 2.275153
H 4.056688 -1.531026 -0.554584
H 0.401901 0.506127 1.361996
H -0.020096 2.538823 1.742649
H -0.158479 3.219970 0.382739
O -3.812830 -0.760967 -1.208600

HNO3-.(H2O)10
H=-1045.613322
O -2.054860 -1.915414 1.799425
O -1.526925 -2.759433 -0.618592
O -3.498857 0.133172 0.806239
O -1.390404 1.886937 0.887864

O -0.012174 -0.045403 1.950467
O -0.885192 1.170053 -1.571045
O 0.762676 3.222820 -2.208894
O 1.071123 3.025293 0.639786
O 2.499973 1.169631 1.982496
O 3.628324 -0.892389 -0.174670
N 2.988647 -1.896682 -0.289792
O 0.595260 -0.950746 -0.552672
O -3.024167 -0.647743 -1.875837
H -2.704263 -1.204146 1.550198
H -3.539576 -0.113420 -0.139686
H -2.555527 -1.455463 -1.603045
H -1.746286 -2.523892 0.339027
H -0.573241 0.747989 1.677127
H -1.174785 1.530720 -0.674787
H -0.268282 0.442153 -1.334137
H 0.410908 -0.660575 0.376763
H 2.079457 1.915717 1.480494
H 1.176644 3.137923 -0.322193
H 2.922538 0.628306 1.305842
H -1.265868 -1.404339 2.070831
H -2.321906 0.032167 -1.943285
H -1.860492 2.625311 1.284108
H -0.018398 -1.696756 -0.698145
H 0.862098 0.316527 2.203820
H 0.158652 2.442613 -2.104689
H 0.168529 3.977073 -2.270576
H 0.160401 2.639903 0.743589
H -2.768280 0.803129 0.846079
H -1.543738 -3.717569 -0.686252
O 3.020334 -2.795102 -1.073633

HNO3-.(H2O)11
H=-1122.067800
O 0.825077 -2.704350 -0.044686
O -3.029757 -0.689696 -1.921519
N -2.850554 0.305494 -1.282580
O 0.164031 -0.237250 -0.925096
O -1.578152 -0.653473 1.037805
O -1.995221 -3.140865 -0.019756
O -2.300606 1.821328 1.918439
O 0.210982 2.739017 1.643927
O 0.357177 2.537970 -1.065137
O 0.614135 -1.584841 2.515721
O 1.906326 0.419204 1.189330
O 3.141325 2.253806 -0.882723
O 2.730776 -0.177807 -2.081321
O 3.499990 -1.803908 0.088037
H 0.609825 -1.810569 -0.430039
H -0.029667 -3.170782 -0.067387
H -2.310558 -2.875978 -0.890300
H -2.003815 -2.302406 0.492558
H -0.932123 -0.408013 0.310885
H -1.923827 0.189083 1.415954
H 1.803295 -0.217334 -1.759008
H 3.186359 -0.829228 -1.513334
H 3.193190 -1.045345 0.614220
H 2.724407 -2.390626 0.056831
H 1.519322 -0.228022 1.828114
H 1.364414 0.297965 0.386310
H -1.382252 2.205656 1.921163
H -2.737467 2.228707 1.162917
H -0.276123 -1.267153 2.264652
H 0.813939 -2.189661 1.772911
H 0.227452 1.560970 -1.070329
H 1.328900 2.633909 -1.149839
H 3.139412 1.417113 -1.405513
H 3.083330 1.942819 0.030659
H 0.218509 2.837083 0.659839
H 0.859420 2.025338 1.775878
H -0.298342 -0.399065 -1.753117
O -3.574048 1.178000 -0.912517

NO
H=-129.931947
O 0.000000 0.000000 0.535356
N 0.000000 0.000000 -0.611835

NO.(H2O)
H=-206.369701
O -1.912590 -0.182635 -0.000595
O 1.601254 -0.353232 -0.000205
N 0.842935 0.506833 0.000393
H -1.703239 0.372309 -0.759776
H -1.706614 0.366803 0.763427

NO.(H2O)2
H=-282.815722
O -0.655235 1.571858 0.130009
O -1.866783 -0.957722 -0.171262
H -0.827222 2.176382 -0.598285
H -1.276460 0.833087 0.009084
H -2.344114 -1.350858 0.567005
H -0.964895 -1.299879 -0.113633
N 1.223768 -0.568401 0.336783
O 2.127808 -0.161627 -0.236454

NO.(H2O)3
H=-359.269657
O 0.281210 -1.090885 0.952583
O 2.308504 -0.459798 -0.781788
O 0.871525 1.577336 0.412633
H 1.013367 -1.183387 0.307894
H 0.526396 -1.610520 1.723975
H 2.368306 -0.526251 -1.739134
H 1.994264 0.445070 -0.578556

H 0.103177 2.003028 0.018720
H 0.511295 0.788039 0.862323
N -2.039324 0.449458 -0.026267
O -2.491430 -0.409427 -0.634847

NO.(H2O)4
H=-435.727625
O 0.469793 1.869748 0.574223
O -0.461467 -0.387889 1.749761
O 2.058843 0.363835 -1.009945
O 0.931611 -1.872947 -0.000725
H 0.097624 1.138874 1.122295
H -0.285627 2.270903 0.132978
H 1.542882 1.024617 -0.486447
H 2.986710 0.596433 -0.912456
H -0.327844 -0.642871 2.667126
H 0.036863 -1.038621 1.192510
H 0.337340 -2.197040 -0.685518
H 1.447998 -1.144372 -0.423860
O -2.062877 0.542140 -0.832519
N -1.903310 -0.589573 -0.921856

NO.(H2O)5
H=-512.179278
O -2.408439 1.333085 -0.304303
O -2.362621 -1.340292 -0.508380
O 0.031262 -2.112859 0.419403
O 1.139673 -0.002788 1.672688
O 0.049563 2.108283 0.454928
H -2.875984 1.687553 -1.065509
H -3.012770 -1.848138 -0.014054
H 0.707696 -2.339766 -0.226799
H 1.168974 0.111480 2.626634
H 0.545479 2.300342 -0.346934
H -2.442656 0.346156 -0.383782
H -1.476342 -1.660885 -0.201292
H 0.426648 -1.384888 0.958021
H 0.734774 0.819696 1.292223
H -0.871329 1.890170 0.163906
N 2.471194 -0.574286 -0.977837
O 2.275206 0.526856 -1.229029

NO.(H2O)6
H=-588.632616
O -0.902696 -1.512577 1.318012
O -2.132526 -1.042240 -0.963807
O -3.014266 1.397142 -0.168673
O -0.691540 1.314105 1.168373
O 1.502377 -1.944402 0.069212
O 0.432214 0.229759 -1.197667
H -0.783636 -0.581737 1.576470
H -0.004943 -1.816943 1.064340
H -0.405476 -0.186450 -1.462514
H 0.192298 0.750035 -0.408077
H 1.275184 -1.172265 -0.497981
H 2.415868 -1.817461 0.341296
H -1.579476 1.510024 0.778380
H -0.451842 2.048912 1.740258
H -3.119279 1.990829 -0.918401
H -2.883651 0.496640 -0.545481
H -1.731239 -1.348829 -0.095898
H -2.591076 -1.793451 -1.351534
O 3.482813 0.503616 -0.193942
N 2.893751 1.479640 -0.067561

NO.(H2O)7
H=-665.091580
O 0.730479 1.263754 1.715861
O 0.717606 -1.336836 1.592451
O -0.152014 -1.711787 -0.967455
O -1.525178 1.822657 0.273944
O -0.028148 0.978738 -1.762958
O 2.685503 -1.367369 -0.351524
O 2.479195 1.297291 -0.502354
H 1.406732 1.491137 1.050821
H -0.118854 1.590534 1.345795
H -2.013175 2.638037 0.127303
H -1.062782 1.596740 -0.576348
H -0.065275 0.012562 -1.614956
H 0.877669 1.233362 -1.510362
H 3.291146 1.766054 -0.716762
H 2.689181 0.326479 -0.488139
H 2.184435 -1.512043 0.476085
H 2.109338 -1.748420 -1.028189
H -1.038911 -2.057701 -1.111185
H -0.034102 -1.655170 0.006135
H 0.685976 -0.339678 1.741514
H 0.540114 -1.770568 2.431720
O -2.984736 -1.067819 0.085213
N -3.547592 -0.085766 -0.114122

NO.(H2O)8
H=-741.531382
O 3.076463 1.034923 -0.677133
O 0.391933 2.480781 -0.540205
O 0.059773 -2.435121 0.813742
O 1.760830 -0.567728 1.592931
O -1.188250 0.238466 -1.363380
O -0.231685 1.282455 2.082109
O -1.976352 -0.542012 1.128182
O -3.926622 0.528948 -0.589485
O 0.421749 -1.956529 -1.904095
H 1.330004 -1.635232 -1.908694
H -0.136911 -1.150487 -1.909122
H 0.141874 -2.460182 -0.163297
H -0.805638 -2.023456 0.989486

H 1.328467 2.262472 -0.642259
H 0.290969 3.401321 -0.805951
H -0.708268 1.073708 -1.233898
H -1.343639 -0.102877 -0.449524
H -2.878067 -0.245921 0.916100
H -1.515621 0.167973 1.637172
H 0.545635 0.683530 2.050674
H -0.100744 1.883088 1.334163
H 2.343345 -0.912017 2.277289
H 1.164699 -1.327753 1.326299
H -3.157961 0.502687 -1.186433
H -4.654990 0.117828 -1.064628
N 3.007732 -0.106877 -0.786959

NO.(H2O)9
H=-818.007819
O 2.261702 -1.573180 -1.403187
O 2.330721 -1.448294 1.227007
O -0.458660 -1.465498 1.377602
O -0.504475 -1.326910 -1.263456
O -0.315577 1.244204 2.039564
O -1.870008 2.257077 0.143401
O -0.627162 1.353641 -1.975581
O 2.090193 1.217008 -1.359816
O 2.361929 1.340768 1.254519
H 2.378802 -1.615254 -0.408689
H 2.874686 -2.198736 -1.800074
H -0.417108 0.276981 1.949303
H 0.633776 1.411070 1.879714
H 0.416551 -1.562868 -1.490104
H -0.629113 -0.422472 -1.615524
H -2.729553 1.848094 0.289974
H -1.296074 1.956309 0.903636
H -1.107092 -2.095728 1.705454
H -0.556207 -1.431902 0.382986
H 1.166512 1.376088 -1.638879
H 2.266535 0.279017 -1.565524
H 2.292004 1.390289 0.256124
H 3.033912 1.970936 1.530739
H -0.949536 1.760610 -2.785228
H -1.135413 1.758311 -1.210445
H 2.490733 -0.501992 1.413380
H 1.399339 -1.599363 1.478118
N -3.609024 -0.468079 -0.188896
O -3.377362 -1.514170 0.215862

NO.(H2O)10
H=-894.465349
O -0.705395 2.277144 -1.545715
O 1.488532 1.650734 -0.126802
O 1.675589 -0.956864 0.048046
O -0.213416 -2.145876 -1.562212
O -1.802957 -0.186545 -2.272516
O -3.430185 -0.459658 -0.041486
O -1.746190 -2.294536 0.789241
O 0.201465 -1.146356 2.384056
O -0.128658 1.428661 2.216247
O -2.352301 1.990477 0.767772
H 0.163258 2.124640 -1.096026
H -0.601517 3.035879 -2.127568
H 1.572971 0.649028 -0.110738
H 2.386602 1.983125 -0.227852
H 1.114595 -1.437373 -0.596723
H 1.304801 -1.170448 0.928772
H -0.077668 -2.780192 -2.272148
H -0.787767 -1.404378 -1.921199
H -2.531235 -0.242957 -1.621477
H -1.409961 0.699005 -2.146944
H -1.932225 2.249447 -0.070070
H -2.845597 1.173232 0.561900
H 0.528004 1.712437 1.557079
H -1.000156 1.663777 1.811113
H 0.038653 -0.150189 2.404254
H 0.438965 -1.420975 3.274577
H -1.205796 -2.469971 -0.003935
H -1.117452 -1.957766 1.460764
H -2.853799 -1.182070 0.348608
H -4.346374 -0.737002 0.050459
N 4.318018 0.307036 -0.028541
O 4.880463 -0.467995 -0.657014

NO.(H2O)11
H=-970.916647
O 0.271335 1.342662 -1.517374
O -1.146070 -0.908696 -0.944671
O -1.844098 -0.149016 1.432122
O 0.456860 -0.511918 2.709091
O -1.542033 3.349719 -1.375641
O -1.249184 2.646898 1.229736
O 1.367958 1.532635 1.042591
O 3.636700 0.303687 0.082750
O 2.430592 -0.220464 -2.188215
O 1.132231 -2.485089 -1.132349
O 2.326493 -1.926582 1.150942
H -0.315221 2.113145 -1.692050
H 0.602163 1.465049 -0.601831
H 2.932460 -1.196654 0.915622
H 1.693667 -1.553383 1.791365
H -1.566615 3.213231 -0.398200
H -1.448839 4.295022 -1.523465
H 1.127424 0.843299 1.692131
H 2.291603 1.335678 0.788403
H -0.283588 2.559796 1.281256
H -1.580973 1.736148 1.338288
H -2.732407 -0.472271 1.615362
H -1.601054 -0.491023 0.510878

H -0.458028 -1.599757 -1.022143
H -0.725199 -0.095193 -1.309336
H -0.474032 -0.474503 2.368258
H 0.422713 -0.418234 3.665780
H 1.201442 -3.411194 -1.382570
H 1.580066 -2.375593 -0.244647
H 2.009274 -1.088549 -2.040147
H 1.688492 0.416984 -2.219291
H 4.571623 0.511148 -0.005020
H 3.273183 0.128983 -0.838189
O -4.131044 -1.568399 -0.121581
N -3.698011 -2.383661 -0.804239

NO2-
H=-205.216249
N 0.000000 0.462518 0.000000
O 1.072090 -0.202433 0.000000
O -1.072090 -0.202270 0.000000

NO2-(H2O)
H=-281.685172
N -1.283941 -0.018383 0.000017
O -0.653624 1.071312 -0.000156
O -0.593913 -1.075192 -0.000081
H 1.416593 0.766215 0.000076
O 2.027704 0.008797 0.000201
H 1.329654 -0.676878 0.000092

NO2-(H2O)2
H=-358.142565
O -2.332176 0.280628 0.080207
N -1.107704 0.127073 0.016269
O -0.688780 -1.069692 -0.127689
O 1.989218 -1.109232 -0.006091
O 1.338820 1.746787 -0.032305
H 0.993557 -1.131784 -0.126515
H 2.082697 -0.897544 0.928259
H 1.806392 0.925781 -0.252087
H 0.414623 1.426104 0.023481

NO2-(H2O)3
H=-434.603007
N 0.223109 -0.779804 0.003883
O 0.389818 0.481893 -0.113294
O 1.244568 -1.469586 0.081781
H 3.139391 -0.221884 0.063682
O 3.239358 0.738091 -0.002668
H 2.293228 0.965961 -0.075995
H -2.136365 1.710885 0.940708
O -2.016494 1.727343 -0.014085
H -1.119682 1.313861 -0.134463
H -2.774654 -0.135847 -0.197993
O -2.753461 -1.092058 -0.030469
H -1.793993 -1.259816 0.006752

NO2-(H2O)4
H=-511.065670
O 0.004901 2.090425 -0.085123
O 1.493885 -0.018940 -1.098303
N 2.199684 -0.344312 -0.095308
O 1.701731 -0.094723 1.027076
O -0.992378 -1.492999 -1.242935
O -2.443969 0.737142 -0.136481
H 0.464949 1.406204 -0.625958
H 0.342778 1.854664 0.789299
H -0.140214 -1.038276 -1.372524
H -0.987874 -1.676552 -0.284670
H -2.152103 0.032839 -0.745034
H -1.691804 1.365007 -0.146414
O -0.963178 -1.035881 1.580389
H -1.571229 -0.353487 1.238653
H -0.070227 -0.660407 1.456833

NO2-(H2O)5
H=-587.527132
O 2.538586 -0.585691 -0.033672
O 0.968219 0.623519 -2.011970
O -0.806498 -1.491413 -0.976571
N -0.214008 -1.595994 0.119295
O -0.851537 -1.220164 1.143327
O -2.630513 0.573473 -0.004687
O -0.289226 2.195546 -0.110325
H 1.847789 -1.263384 0.075497
H 2.196475 -0.080188 -0.803054
H 0.385906 -0.151537 -1.961245
H 0.529110 1.279592 -1.420490
H -1.173011 1.779165 -0.058217
H 0.189020 1.852288 0.673970
H -2.459133 0.106976 -0.834917
H -2.186839 -0.051539 0.610797
O 1.070285 0.802756 1.939361
H 0.412320 0.086148 1.965380
H 1.761879 0.430232 1.353500

NO2-(H2O)6
H=-663.982335
O 1.424873 -1.975831 0.489403
O 0.526860 -0.198885 -1.509555
O -1.275212 -1.768165 1.165637
O 2.084853 0.592017 1.395683
O -2.209860 -1.059842 -1.403070
O 3.074658 0.962066 -1.102644
O -0.576551 1.000187 0.735374
H 3.676343 0.213606 -1.167734
H 2.778874 0.948194 -0.153971
H 1.204297 -1.529083 -0.352427

H 1.365005 0.287337 -1.629576
H 0.530711 -2.129255 0.861916
H -1.693241 -1.787647 0.280519
H -2.362396 -0.173124 -1.010593
H -1.338845 -0.965255 -1.817598
H 1.925710 -0.369177 1.264723
H 1.183695 0.970802 1.366419
H -1.112018 -0.813929 1.270986
H 0.072618 0.268903 -0.771794
O -2.459541 1.491733 -0.120922
N -1.564485 1.818910 0.665697

NO2-(H2O)7
H=-740.441965
O -0.033172 -0.790960 1.725534
O -0.071438 1.885732 0.882178
O -1.146920 -1.819938 -1.324727
O -0.276357 0.732914 -1.789863
O 2.412129 -0.071344 -1.651573
O -2.741062 -0.850466 0.744695
O 2.651165 1.402180 0.704794
O -2.618775 1.817899 -0.310140
H 2.444905 0.597387 1.211530
H 2.719892 1.041829 -0.206828
H 0.902560 1.994767 0.805005
H -0.156177 1.069319 1.410767
H -0.416569 -2.054120 -0.716081
H -1.892777 -1.627402 -0.719725
H -1.881956 -0.910548 1.201859
H -2.817797 0.082124 0.463144
H -0.180474 1.067170 -0.878719
H -0.623212 -0.189637 -1.698026
H 1.523114 0.246011 -1.916736
H 2.218619 -0.871951 -1.136848
H -1.834320 2.121132 0.182018
H -2.235531 1.567977 -1.164270
N 1.101150 -1.050384 1.241295
O 1.139639 -1.903688 0.328332

NO2-(H2O)8
H=-816.898574
O -1.524061 -1.254463 1.729183
O 1.066019 -1.111957 1.314571
O 0.247351 1.168947 -1.518326
O 2.994431 1.551911 -0.755658
O 1.073650 1.618807 1.290593
O -1.725840 1.592281 1.602396
O -2.569474 1.498576 -1.058335
O -2.703565 -1.271042 -0.849477
O -0.219058 -1.487409 -1.993848
H -0.537460 -1.315644 1.558464
H -1.703989 -1.809666 2.492615
H -0.776192 1.810220 1.546759
H -1.738612 0.633488 1.776054
H 1.831700 1.895036 0.739862
H 1.233197 0.660834 1.443736
H 0.335872 -1.929883 -1.332388
H -1.142581 -1.535993 -1.648069
H 0.134074 0.212149 -1.744158
H 0.286332 1.210099 -0.547293
H -2.387452 1.664702 -0.108014
H -1.682022 1.546296 -1.458844
H -2.434227 -1.351236 0.082934
H -2.801458 -0.301137 -0.988744
H 3.119601 0.626718 -0.481031
H 2.214942 1.506905 -1.338928
O 2.705375 -1.257031 -0.036489
N 1.612807 -1.701693 0.315739

NO2-(H2O)9
H=-893.355678
O -1.287751 2.464094 0.227116
O -2.006261 0.434463 -1.754370
O -3.519040 -0.963693 0.339899
O 1.466383 2.531325 -0.060250
O 2.531305 0.626245 1.490862
O 0.579132 -0.862409 2.376832
O 0.900226 -2.479642 0.126563
O 3.039034 -1.170050 -0.635097
O 1.629211 0.741378 -2.205610
O -1.887268 0.515375 2.102247
H 3.730253 -1.773046 -0.920489
H 2.244199 -1.732405 -0.380885
H 2.929075 0.068951 0.797797
H 2.154496 1.408016 0.996959
H 2.206562 0.079971 -1.784662
H 0.731421 0.355465 -2.143834
H 0.142844 -2.213904 -0.447226
H 0.708869 -2.065727 0.992297
H 0.496427 2.599243 0.095722
H 1.542976 2.041925 -0.907542
H -2.496399 -0.064716 1.600160
H -1.057574 0.011961 2.198511
H -1.546515 1.991344 -0.579590
H -1.515989 1.838755 0.953255
H -2.792141 -1.393779 -0.160848
H -3.688266 -0.211882 -0.244330
H 0.755843 -1.077381 3.297000
H 1.321091 -0.242946 2.080561
N -1.039143 -0.341625 -1.692467
O -1.269117 -1.490646 -1.207642

NO2-(H2O)10
H=-969.812833
O 0.604319 -2.434858 -0.585222
N 0.838116 -0.073102 1.912829

O 2.761247 1.338921 -0.683395
O -2.516129 -1.141741 1.392250
O -2.911913 1.286937 0.538824
O -0.522001 2.467905 1.363459
O 0.647707 3.070948 -1.027735
O 3.261989 -1.636935 -0.406859
O -2.216608 -2.252884 -1.094842
O -2.185871 0.367836 -2.057590
O 0.553511 0.048145 -1.929178
H 0.507365 -2.144979 0.345431
H 1.573195 -2.434313 -0.720690
H 2.940094 -1.291611 0.450125
H 3.361019 -0.832299 -0.934709
H 2.603402 1.073027 0.240296
H 2.199248 2.135702 -0.809855
H -1.596918 -1.146836 1.731603
H -2.474705 -1.662543 0.552751
H -2.269943 -1.392104 -1.567828
H -1.277137 -2.499742 -1.119685
H -1.209102 0.427681 -2.066020
H -2.470217 0.810746 -1.237832
H 0.223552 2.941011 -0.143480
H 0.195831 2.435540 -1.597061
H 1.361255 0.449324 -1.553480
H 0.492472 -0.831976 -1.507137
H -0.060162 1.683955 1.718764
H -1.414375 2.143666 1.129096
H -2.818245 0.354768 0.917589
H -3.790476 1.597596 0.773990
O 2.053455 -0.108076 1.619212
O 0.227424 -1.169311 1.872117

NO2-(H2O)11
H=-1046.265570
O 0.170850 -1.167581 0.401401
O 3.497910 -2.022854 0.447323
O -2.418804 -2.016790 -1.540921
O 1.404220 1.077845 1.418312
O -0.512372 1.579717 3.304102
O 0.336568 2.880574 -0.285156
O 1.817893 1.732156 -2.295477
O -3.527072 0.022892 -0.005909
O -2.198495 1.944158 -1.138628
O -1.984508 -0.538321 2.165871
O 3.567583 0.693083 -0.387049
O -0.446582 0.000329 -2.098247
H -2.763936 1.248264 -0.664481
H -2.792563 2.467875 -1.682878
H -3.272187 -0.768280 -0.533906
H -3.105474 -0.129978 0.873573
H -1.278527 -0.904090 1.602756
H -1.556210 0.191240 2.658215
H -1.053279 0.740698 -1.894228
H -0.204588 -0.356671 -1.221368
H 1.162192 1.023263 -2.429622
H 2.526831 1.330620 -1.746396
H -0.580508 2.650324 -0.520333
H 0.877096 2.633778 -1.074007
H 1.008012 1.761542 0.815988
H 1.064803 0.220117 1.079077
H 2.991630 0.944946 0.359567
H 3.648440 -0.274444 -0.287569
H 3.602261 -1.834780 1.385897
H 2.550958 -2.270370 0.352203
H -1.800850 -2.502430 -0.963326
H -1.824027 -1.428448 -2.039053
H 0.292502 1.396061 2.762192
H -0.847474 2.407862 2.944653
N 0.662020 -2.342638 0.229606
O -0.117096 -3.203787 -0.158647

NO2
H=-205.140890
N 0.000000 0.322431 0.000000
O 1.099453 -0.141071 0.000000
O -1.099453 -0.141056 0.000000

NO2.(H2O)
H=-281.579082
N 1.085231 0.000000 -0.222701
O 0.752922 -1.097634 0.104887
O 0.752927 1.097635 0.104922
H -1.785635 -0.765002 0.421045
O -2.008987 -0.000060 -0.120115
H -1.785874 0.765470 0.420311

NO2.(H2O)2
H=-358.027057
O -2.217788 0.248042 -0.027444
N -1.187445 -0.289058 -0.264079
O -0.581778 -1.183769 0.260386
O 2.323708 -0.605086 -0.187724
O 0.680198 1.630360 0.187768
H 1.528919 -1.145932 -0.082947
H 2.940977 -0.907297 0.486361
H 1.424502 1.020306 0.034607
H 0.783004 2.339948 -0.453355

NO2.(H2O)3
H=-434.480294
O 2.392259 -0.733951 -0.919112
O 1.534555 1.482962 0.462328
O 0.457772 -1.033863 0.999180
N -1.720304 0.011927 -0.472751
O -1.456640 1.141947 -0.182450
H 2.294134 0.194497 -0.618619

H 3.334245 -0.927227 -0.910532
H 0.839249 2.009237 0.053107
H 1.056977 0.793735 0.960413
H 0.467369 -1.732406 1.659624
H 1.138814 -1.256702 0.330658
O -2.564029 -0.752672 -0.130620

NO2.(H2O)4
H=-510.938764
O 1.105907 -1.939204 0.019882
O -1.909189 -1.108421 -0.061612
N -1.452163 -0.007633 -0.022228
O 0.999893 0.010665 -1.858566
O 0.925848 -0.000022 1.881515
O 1.078782 1.951870 0.008419
H 1.135418 -1.338530 -0.762154
H 0.268985 -2.410018 -0.056396
H 1.036387 -0.794895 1.301924
H 1.514037 -0.116048 2.633322
H 1.619696 0.134019 -2.583329
H 1.073873 0.807288 -1.275730
H 0.235740 2.415498 0.055053
H 1.086263 1.351970 0.791479
O -1.926897 1.085630 0.016790

NO2.(H2O)5
H=-587.390303
O 2.542395 1.263134 0.560494
O 2.420997 -1.417433 0.651213
O 0.218986 -2.150508 -0.697187
N -1.749268 -0.002288 0.814878
O -2.315502 -1.051149 0.827458
O -0.757378 0.068353 -1.876894
O 0.356931 2.183314 -0.711147
H 2.859903 1.577349 1.411358
H 3.151291 -1.907409 0.261768
H -0.535341 -2.472029 -0.192966
H -0.848456 0.185019 -2.826712
H -0.261310 2.468673 -0.030285
H 2.537797 0.273930 0.605394
H 1.604857 -1.727992 0.183574
H -0.120500 -1.377872 -1.209669
H -0.314507 0.884330 -1.521413
H 1.184472 1.919007 -0.238071
O -2.093095 1.128415 0.977673

NO2.(H2O)6
H=-663.844783
O 0.090816 1.595964 -0.365725
N -3.238256 0.116671 0.271943
O -3.869075 -0.862738 0.041908
O -0.579766 -0.920440 0.135770
O 2.306451 1.445502 1.196887
O 1.606243 -1.263568 1.753131
O 1.777558 -1.903144 -1.057381
O 2.471150 0.657191 -1.544252
H -0.254727 0.672163 -0.205296
H -0.684116 2.157209 -0.467997
H 1.611614 1.112404 -1.500302
H 2.897941 0.942004 -0.720938
H 2.555504 2.136431 1.817909
H 1.446900 1.702758 0.794701
H 2.104226 -1.014023 -1.345400
H 2.148486 -2.553063 -1.661285
H 1.975967 -0.363213 1.727483
H 1.952374 -1.696181 0.955129
H -0.027951 -1.067396 0.937541
H -0.087479 -1.437675 -0.525687
O -2.924747 1.075218 -0.374020

NO2.(H2O)7
H=-740.302362
O 0.757298 1.182665 1.741635
O 0.932010 -1.409600 1.522213
O 0.429970 -1.671143 -1.155716
O -2.440966 -1.225483 -0.208018
N -3.179826 -0.287615 -0.285403
O -1.279471 1.736171 -0.024235
O 0.570073 1.049467 -1.807602
O 3.142771 -1.248741 -0.124705
O 2.831424 1.407777 -0.162900
H 1.517923 1.471039 1.203494
H -0.038218 1.481826 1.249892
H -1.677149 2.598936 -0.174803
H -0.662566 1.560882 -0.785728
H 0.558287 0.076080 -1.701222
H 1.412145 1.331785 -1.406812
H 3.644457 1.918282 -0.220446
H 3.081104 0.445998 -0.167916
H 2.537994 -1.465041 0.613349
H 2.694607 -1.619699 -0.896849
H -0.444832 -1.971283 -1.423881
H 0.415036 -1.658141 -0.174089
H 0.813502 -0.426781 1.707687
H 0.644726 -1.898601 2.298274
O -3.972887 0.199889 0.453938

NO2.(H2O)8
H=-816.765670
O 0.523943 1.775907 0.119896
O 3.501219 1.096680 -0.196325
N 3.757721 0.022666 0.265229
O 1.055186 -0.826910 -0.016071
O -0.801404 -1.320944 -2.022178
O -2.586247 -1.773338 -0.142366
O -0.796402 -1.422876 1.976483

O -1.449693 1.124785 1.988869
O -3.365679 0.907749 -0.027405
O -1.472198 1.393407 -1.789931
H 1.329222 2.303212 0.120256
H 0.816518 0.824202 0.072019
H -4.197374 1.364352 -0.184591
H -2.731239 1.164936 -0.759206
H -1.523037 -1.588709 -1.380185
H -0.809828 -1.947127 -2.752129
H -2.243418 1.173813 1.420450
H -0.743822 1.562017 1.474271
H -1.198696 0.509053 -2.100679
H -0.723444 1.713916 -1.249245
H 0.566361 -1.119226 -0.811761
H 0.561020 -1.204134 0.738716
H -0.732396 -1.829351 2.845726
H -1.042173 -0.459315 2.105459
H -2.058424 -1.840366 0.677087
H -3.071419 -0.928924 -0.062531
O 4.328541 -0.936586 -0.142254

NO2.(H2O)9
H=-893.218045
O 1.951796 1.136588 -0.269201
O -0.312230 2.370355 0.253409
O 0.982444 -1.304208 -0.593571
O 3.880342 -1.226658 0.408412
N 4.494208 -0.204315 0.440654
O -1.620890 0.906654 2.226581
O -0.846482 -1.502703 1.512808
O -2.119226 1.482949 -1.678462
O -1.372310 -1.013302 -2.061788
O -3.722169 0.576108 0.420909
O -3.097065 -1.934029 -0.077442
H 2.421288 1.635889 -0.943992
H 1.129392 1.662446 -0.042389
H -1.623363 -1.864641 1.044990
H -0.129814 -1.480884 0.847286
H -0.733791 1.988247 1.048598
H -0.937761 2.189959 -0.475793
H 1.405222 -0.404187 -0.526829
H 1.705344 -1.939215 -0.615352
H -3.458040 -1.021308 0.124672
H -3.831476 -2.554372 -0.060783
H -0.489444 -1.195097 -1.684820
H -2.001494 -1.511176 -1.504122
H -1.592500 1.043088 3.178108
H -1.269672 -0.013722 2.040524
H -1.802797 0.560643 -1.910971
H -2.364775 1.921613 -2.498317
H -3.151255 0.812865 1.177837
H -3.331789 1.045698 -0.342034
O 4.850449 0.577539 -0.384303

NO2.(H2O)10
H=-969.675750
O -1.176692 2.214257 -1.605422
O 1.044758 1.824122 -0.143934
N 4.128492 -0.073505 -0.530642
O 4.011024 1.034591 -0.092361
O 1.458020 -0.760924 0.106420
O -0.294343 -2.145225 -1.492852
O -2.041648 -0.355839 -2.270834
O -3.671942 -0.714901 -0.053308
O -1.839231 -2.367492 0.845596
O -0.020363 -1.005501 2.426745
O -0.581820 1.521398 2.182745
O -2.826565 1.843196 0.696085
H -0.304426 2.153518 -1.141498
H -1.132239 2.960920 -2.210051
H 1.215345 0.836488 -0.099582
H 1.911841 2.231948 -0.240006
H 0.946473 -1.305258 -0.529369
H 1.092339 -0.977770 0.989259
H -0.096841 -2.786291 -2.181902
H -0.928197 -1.467407 -1.877527
H -2.772008 -0.460752 -1.628242
H -1.731996 0.564813 -2.165455
H -2.420404 2.117846 -0.143517
H -3.242746 0.980279 0.506910
H 0.054902 1.846609 1.523113
H -1.465808 1.665887 1.762319
H -0.274153 -0.027977 2.417209
H 0.228318 -1.234114 3.327192
H -1.275297 -2.514466 0.063216
H -1.251861 -1.959040 1.514571
H -3.039063 -1.372097 0.363042
H -4.561013 -1.070461 0.035788
O 4.707225 -1.046199 -0.170252

NO2.(H2O)11
H=-1046.127955
O 0.332255 1.002471 -1.675416
O -1.041169 -0.973072 -0.415826
O -3.845175 -1.068395 0.997286
N -3.870511 -0.927174 -0.192407
O -1.262309 0.325813 1.829187
O 1.211619 0.047670 2.749960
O -1.389545 3.089826 -1.660535
O -0.615706 2.959814 0.932618
O 1.882810 1.638905 0.555188
O 3.886161 0.066823 -0.493055
O 2.269158 -0.823057 -2.360016
O 1.096142 -2.725777 -0.642521
O 2.699126 -1.791939 1.228570
H -0.258914 1.754124 -1.907961

H 0.827679 1.290041 -0.878591
H 3.284166 -1.175807 0.745492
H 2.208076 -1.247324 1.870910
H -1.221609 3.176409 -0.691052
H -1.321000 3.971863 -2.036665
H 1.737455 1.121546 1.371260
H 2.734544 1.319293 0.195918
H 0.340573 2.822702 0.836513
H -0.946085 2.116020 1.294944
H -2.128440 0.127726 2.201395
H -1.198675 -0.222376 0.984456
H -0.412371 -1.721510 -0.459063
H -0.650502 -0.284934 -1.005095
H 0.235838 0.088919 2.576582
H 1.358984 0.313162 3.662654
H 1.098161 -3.685695 -0.702888
H 1.700802 -2.467041 0.111567
H 1.848966 -1.607767 -1.959250
H 1.559463 -0.150305 -2.398293
H 4.796532 0.184867 -0.779654
H 3.356936 -0.265697 -1.280223
O -4.205491 -1.620083 -1.096203

NO3
H=-280.450396
N -0.000001 0.000007 0.000000
O -0.582856 1.117775 0.000000
O 1.259464 -0.054129 0.000000
O -0.676608 -1.063652 0.000000

NO3.(H2O)
H=-356.913133
O 2.512583 -0.000389 0.000354
O -0.138709 -1.094581 -0.000278
N -0.774462 0.000046 -0.000088
O -2.022743 -0.000804 0.000067
O -0.140164 1.095369 -0.000115
H 1.864532 -0.725456 0.000138
H 1.868969 0.728371 0.000251

NO3.(H2O)2
H=-433.371957
O -2.937696 -0.967054 -0.000755
O 1.087060 1.226574 -0.002284
N 0.000277 0.596726 -0.000574
O -1.087087 1.225722 0.003139
O 2.936776 -0.968177 0.001540
H -2.088853 -1.430570 -0.000809
H -2.596720 -0.055051 0.001001
H 2.085151 -1.426781 0.001433
H 2.600772 -0.054468 -0.000121
O 0.000662 -0.668342 -0.001326

NO3.(H2O)3
H=-509.832341
O -1.816065 -0.989559 1.308862
O -1.711283 -0.551785 -1.560100
O 1.207059 1.169222 0.070817
N 1.582262 -0.034047 0.012832
O 2.783348 -0.322413 -0.019864
O 0.701724 -0.960179 -0.012406
H -2.157607 -1.103305 0.406024
H -0.855465 -0.999740 1.136560
H -1.805688 0.354063 -1.207614
H -0.808525 -0.778257 -1.272253
O -1.549290 1.679530 0.196605
H -0.574590 1.578701 0.142686
H -1.797903 0.988345 0.833469

NO3.(H2O)4
H=-586.289626
O 1.413555 1.772435 -0.598296
O -1.378828 2.215461 -0.101246
O -0.959378 -0.230927 1.154115
N -1.179255 -0.915919 0.098554
O -2.088863 -0.569694 -0.680667
O -0.466655 -1.917092 -0.136924
O 1.854087 -0.071565 1.539584
H 0.475561 2.036249 -0.536459
H 1.616769 1.391145 0.277539
H 2.153512 -0.585777 0.768754
H 0.897676 -0.267162 1.577290
H -1.852638 1.698482 -0.766845
H -1.299776 1.541018 0.603711
H 1.192835 -1.398959 -1.034605
O 2.035260 -0.948714 -1.214866
H 1.797420 -0.002791 -1.272863

NO3.(H2O)5
H=-662.748162
O 0.875843 1.284498 -1.664594
O 2.960411 -0.113878 -0.457066
O -0.170045 2.572177 0.606811
O -0.672966 -0.663039 -0.291686
N -1.939696 -0.460186 -0.124906
O -2.314197 0.688537 0.202602
O -2.730779 -1.386241 -0.291350
O 1.165774 0.235769 1.681402
H 0.637530 1.960316 -0.997172
H 0.251519 0.571789 -1.437348
H 0.438286 -0.049833 1.096104
H 1.938659 0.235994 1.078369
H 0.375681 2.030407 1.203135
H -0.967242 2.021540 0.479698
H 2.594301 -1.014595 -0.497749
H 2.356643 0.426133 -1.013584

O 1.382162 -2.466001 0.212439
H 1.460674 -2.044787 1.079039
H 0.522203 -2.130232 -0.104616

NO3.(H2O)6
H=-739.209153
O -1.174107 1.913516 -1.380735
O -0.401407 2.192881 1.213376
O -1.472791 -0.119210 1.894913
O -2.622970 -0.366346 -0.728459
O -0.562444 -2.078444 -1.429685
O 0.184088 -2.336928 1.268695
O 2.341851 -0.833781 0.140349
N 1.864876 0.237297 -0.295433
O 1.130034 0.221889 -1.328837
O 2.104220 1.324580 0.279962
H -1.994276 -1.039060 -1.078615
H -2.418463 -0.339708 0.222115
H -0.368069 -2.400329 -0.525919
H 0.123675 -1.397356 -1.562348
H -0.365358 -1.590125 1.573761
H 1.032426 -1.930164 1.018084
H -1.885807 -0.030698 2.758113
H -1.041442 0.766294 1.695393
H -0.315771 1.478855 -1.540072
H -1.794306 1.158373 -1.261180
H -0.783532 2.278998 0.304437
H 0.544997 2.038579 1.027630

NO3.(H2O)7
H=-815.665104
O -2.993928 0.488588 -0.771901
O -2.397546 -0.030132 1.926438
O -0.892189 2.336778 -0.849582
O 0.060723 1.442084 1.511328
O 1.378554 1.112338 -1.903694
O 2.733326 1.501291 0.508889
O 2.790044 -1.301277 0.907086
O 0.761118 -1.673527 -1.201318
N -0.227574 -1.658262 -0.433361
O -1.369341 -1.914508 -0.841943
O -0.052842 -1.370559 0.803600
H -2.984183 0.391691 0.203264
H -2.603156 -0.348606 -1.065030
H -1.828347 -0.764980 1.640206
H -1.750348 0.678197 2.087175
H -1.671719 1.755056 -0.973141
H -0.167243 1.959464 -1.393049
H 1.214261 0.158175 -1.853826
H 1.951450 1.306333 -1.127992
H 0.152921 0.513010 1.241060
H -0.311367 1.882028 0.701033
H 1.924444 1.705725 1.015029
H 2.920243 0.569511 0.739291
H 2.759911 -1.555919 -0.024416
H 1.842818 -1.370464 1.132705

NO3.(H2O)8
H=-892.120494
O 2.760514 1.058194 1.717312
O -0.136513 1.535118 1.543714
O -0.705637 2.828437 -0.892570
O -2.717614 0.426132 1.526410
O -1.604114 -2.139898 1.479706
O -2.069918 0.230522 -1.269388
N -0.973968 -0.326732 -1.511735
O -0.007075 0.359432 -1.953317
O -0.828844 -1.556369 -1.326645
O 0.932203 -1.042374 1.524591
O 2.818652 0.480568 -1.071137
O 1.938092 -2.153311 -0.787238
H 1.082076 -2.034212 -1.234075
H 2.444187 -1.343850 -1.006284
H 1.900872 0.625149 -1.345806
H 2.867160 0.768238 -0.136061
H 2.036393 1.690001 1.840270
H 2.315358 0.212381 1.911613
H 0.229643 0.646068 1.347668
H -0.229860 2.017312 0.696241
H -0.362687 2.108985 -1.460439
H -1.647071 2.617182 -0.872915
H -1.889412 0.914433 1.717578
H -2.724088 0.375832 0.556294
H -2.113653 -1.321146 1.658647
H -1.549463 -2.166068 0.510653
H 0.093585 -1.518881 1.712142
H 1.326768 -1.515908 0.755118

NO3.(H2O)9
H=-968.575520
O -2.739936 1.563775 1.428071
O -2.836733 1.123970 -1.321659
O -2.531715 -1.573422 -0.914828
O -2.414523 -1.172459 1.816143
O 0.075156 1.669233 0.963654
O 0.084256 1.138910 -1.830133
N 0.774397 0.098038 -1.715060
O 1.989827 0.187485 -1.401237
O 0.259366 -1.031649 -1.903125
O 1.269414 -2.741648 0.040907
O 0.480934 -0.903934 1.992956
O 3.166368 -0.542107 1.111795
H -2.866279 1.527783 0.453289
H -1.809648 1.821402 1.526524
H -1.949014 1.234200 -1.694109
H -2.915319 0.144685 -1.244239

H -0.104981 1.667973 0.007901
H 0.985506 2.032908 1.022283
H -2.626543 -0.211765 1.789845
H -1.496787 -1.210376 2.132705
H 0.264996 -0.047087 1.567200
H 0.552794 -1.580326 1.287580
H -1.619657 -1.551313 -1.239078
H -2.474103 -1.562796 0.069199
H 1.046135 -2.279349 -0.790847
H 2.206044 -2.565272 0.191649
H 2.375324 -0.669924 1.671476
H 2.798188 -0.466991 0.214770
O 2.757154 2.238786 0.566138
H 3.138775 1.447603 0.989031
H 2.597230 1.926858 -0.339221

NO3.(H2O)10
H=-1045.032240
O 3.955662 0.282998 -0.156826
O 2.264756 0.291471 -2.382785
O 2.105338 2.092087 0.920375
O 0.313020 2.039317 -1.086005
O 1.129376 0.222675 2.741250
O -1.422843 0.474441 1.633839
O -3.476156 -1.249446 1.075304
O -1.120285 -2.527070 -0.290459
N -0.437912 -1.649565 -0.828111
O 0.772966 -1.861297 -1.126437
O -0.933736 -0.510661 -1.083599
O 2.360886 -1.821512 1.092622
O -2.232238 2.718377 -0.038791
H 3.494148 0.265613 -1.022681
H 3.611195 -0.489908 0.317968
H 1.679286 -0.440171 -2.120449
H 1.734783 1.088911 -2.211865
H 1.797990 -1.970073 0.301725
H 2.559630 -2.693762 1.445846
H 2.876307 1.585550 0.588277
H 1.775245 1.558447 1.674956
H 1.496837 -0.546303 2.274405
H 0.180716 0.250290 2.504851
H -0.005742 1.121426 -1.090604
H 0.951339 2.106013 -0.323984
H -1.175725 0.321146 0.708302
H -2.202910 -0.119129 1.725334
H -2.049721 2.273416 0.804200
H -1.363082 2.707427 -0.490530
H -2.857398 -1.843824 0.622170
H -3.758824 -0.627304 0.369371
O -3.656087 0.538160 -1.062637
H -3.289871 1.397997 -0.757705
H -2.874098 0.084883 -1.409616

NO3.(H2O)11
H=-1121.494291
O 2.659189 -2.539517 -0.207334
O 3.528870 -0.709631 1.683884
O 3.694872 1.261179 -0.260876
O 2.470982 -0.464154 -2.037585
O -0.135984 -2.120521 0.230642
O -2.515995 -2.162341 1.572926
O -1.927827 0.621330 1.982735
O 0.729514 0.036540 1.728586
O 1.089553 2.326279 0.208651
O -1.468019 -0.699963 -1.911717
N -1.210660 0.485719 -1.584889
O -2.040657 1.135102 -0.888521
O -0.137200 1.014599 -1.942294
O -1.398944 3.295671 0.983083
H 2.610890 -1.899406 -0.952262
H 1.729583 -2.675469 0.036496
H 1.588014 -0.100540 -2.192861
H 2.949884 0.213164 -1.504681
H -0.430946 -1.857643 -0.654876
H -0.966906 -2.285605 0.741896
H 3.338708 -1.458145 1.072424
H 2.648322 -0.472929 2.019589
H 0.564247 -0.722522 1.125899
H 0.968196 0.804389 1.164021
H 2.976919 1.857286 0.004139
H 3.768284 0.612620 0.477177
H 0.713051 2.029361 -0.640549
H 0.378419 2.871667 0.604348
H -0.961985 0.426732 2.060067
H -2.076525 0.635778 1.022923
H -1.639410 2.630539 1.651190
H -1.702221 2.879277 0.160338
H -2.423775 -1.293990 2.005127
H -3.148279 -1.987023 0.846374
O -3.937908 -1.257604 -0.673383
H -3.133781 -1.163847 -1.222032
H -4.159635 -0.339478 -0.480917

NO3-.HNO3
H=-561.468459
O -1.127231 0.823317 -0.457717
N -1.964659 -0.040265 0.073337
O -3.118851 -0.040063 -0.354227
O -1.559898 -0.792235 0.963503
O 1.126812 0.843483 0.434913
N 1.966536 -0.047882 -0.076682
O 1.559806 -0.814741 -0.945291
O 3.112406 -0.033083 0.361341
H 0.042509 0.723608 0.003230

NO3-.HNO3.(H2O)

H=-637.921493
O 0.094819 2.824312 -0.006477
O -1.457958 0.429280 0.895262
N -1.943886 -0.354182 0.078251
O -1.173063 -1.321700 -0.407293
O -3.096496 -0.322339 -0.320323
O 1.123279 -1.339808 0.401283
N 1.914483 -0.413781 -0.067853
O 3.077448 -0.396294 0.320859
O 1.453869 0.397652 -0.890821
H 0.721036 2.176582 -0.363833
H -0.586544 2.256397 0.376568
H -0.103857 -1.226071 -0.025434

NO3-.HNO3.(H2O)2
H=-714.375558
O -1.072355 2.158333 0.552923
O -3.559121 1.165964 -0.558929
O -2.113325 -1.477685 -0.210204
N -0.963236 -1.293132 0.197696
O -0.708040 -0.554544 1.174871
O 0.011371 -1.885388 -0.396777
O 2.252098 -0.927371 0.128736
N 2.334969 0.373579 -0.208292
O 3.384270 0.923401 0.062187
O 1.372710 0.891753 -0.761814
H 1.260087 -1.280040 -0.077393
H -1.042827 1.270721 0.955192
H -0.305617 2.144653 -0.033395
H -2.793378 1.691516 -0.271334
H -3.261258 0.254319 -0.426853

NO3-.HNO3.(H2O)3
H=-790.834074
O 3.253860 0.667469 -0.950099
O 0.871690 2.230324 -0.625299
O 1.828816 -1.783727 -0.342750
N 0.633291 -1.663150 -0.031303
O 0.308394 -0.686785 0.770553
O -0.248697 -2.416248 -0.437591
O 2.246007 1.201656 1.660721
O -2.156897 -0.246910 0.984807
N -2.617497 0.421371 -0.096060
O -1.810855 0.773546 -0.948000
O -3.812954 0.619751 -0.119314
H -1.131956 -0.454470 0.837549
H 2.860990 -0.213455 -0.825958
H 2.485812 1.239169 -1.136468
H 0.086187 1.673292 -0.716858
H 1.181110 2.075347 0.287958
H 1.665221 0.428241 1.545242
H 2.907165 1.071721 0.955854

NO3-.HNO3.(H2O)4
H=-867.298220
O 0.106626 -2.246376 0.887575
O -2.139338 0.410548 -1.613613
N -2.081245 -0.599130 -0.876595
O -2.599531 -0.577309 0.276806
O -1.496714 -1.641099 -1.265214
O 2.570610 -1.147884 0.022018
N 2.605809 0.049112 -0.340030
O 3.337500 0.450677 -1.234288
O 1.835634 0.899855 0.273428
O 0.291931 -0.008214 2.069153
O -1.629638 1.577936 1.589929
O -0.371742 2.438429 -0.674889
H 1.005613 0.341751 1.400822
H -0.569969 -2.210986 0.172557
H 0.964624 -2.101371 0.444590
H -0.529918 0.627386 1.960916
H 0.073424 -0.948991 1.697212
H -1.228167 2.091693 0.845811
H -2.241780 0.956466 1.132563
H -0.893720 1.814940 -1.212455
H 0.505243 2.026748 -0.572882

NO3-.HNO3.(H2O)5
H=-943.760127
O -0.000479 2.029681 -1.362599
O -0.693445 2.069843 1.234466
O 0.570326 0.021102 2.060430
O -3.066582 1.089666 0.113610
N -2.700791 -0.005921 -0.366232
O -3.058547 -1.085999 0.153908
O -1.948198 -0.021828 -1.389466
O -0.679357 -2.048279 1.268465
O 2.913256 0.021855 1.078026
N 2.989360 0.002374 -0.199328
O 1.905568 -0.006633 -0.861289
O 4.080076 -0.007009 -0.751276
H -1.634537 1.865897 1.073159
H 0.770761 1.436018 -1.338098
H -0.755411 1.436557 -1.548428
H 0.048700 0.863301 1.752291
H -0.355516 2.231361 0.313629
H 0.055521 -0.830535 1.765228
H 1.512305 0.021682 1.623730
H -0.338557 -2.229107 0.352605
H -1.620036 -1.846574 1.099622
H -0.747626 -1.473104 -1.521653
O 0.010483 -2.060723 -1.330994
H 0.779615 -1.464072 -1.319428

NO3-.HNO3.(H2O)6
H=-1020.218148

O 2.365077 0.960390 1.396866
O 0.079018 0.508865 2.342196
O -0.542035 -1.874377 1.843111
O -1.840822 1.323762 0.792548
N -1.338846 1.842270 -0.272932
O -0.398640 2.650935 -0.143026
O -1.794308 1.527127 -1.379228
O -2.861059 -1.323206 0.608339
O 2.407372 -1.490185 0.172660
N 1.526004 -1.497421 -0.721629
O 0.607124 -2.365242 -0.672175
O 1.530565 -0.659470 -1.648489
O -1.774296 -1.398399 -1.913453
H -0.638600 0.995926 1.822172
H 2.611834 0.086912 1.023142
H 2.350197 1.540171 0.591849
H -0.117064 -0.516926 2.177444
H 1.029809 0.759117 1.962127
H -1.456467 -1.782571 1.450965
H -0.006252 -2.252810 1.117115
H -2.614388 -1.503843 -0.328365
H -2.782940 -0.353004 0.657725
H -1.692893 -0.433166 -1.961260
H -0.878759 -1.724417 -1.704567
O 2.166458 2.045250 -1.072188
H 1.253603 2.382104 -1.027039
H 2.076179 1.144958 -1.436677

NO3-.HNO3.(H2O)7
H=-1096.668417
O -2.267344 -0.643592 -1.910309
O -0.426353 0.969364 -2.481209
O 1.512337 -0.623411 -1.519950
O 0.787771 2.591615 0.414359
N -0.378522 2.211857 0.625600
O -0.696789 1.584795 1.659875
O -1.278615 2.458768 -0.243977
O 2.612425 0.478432 0.716400
O -3.218816 0.564413 0.126302
O -0.691640 -2.325032 -0.798950
N -0.748938 -2.231176 0.488411
O -1.793036 -1.815634 1.018303
O 0.247910 -2.555189 1.146411
O 1.067200 -0.376570 2.801562
H -0.548714 1.721777 -1.869400
H -1.737455 -1.412149 -1.480673
H -2.760592 -0.178757 -1.128984
H 0.391325 0.486080 -2.179303
H -1.532246 0.044069 -2.231313
H 1.891402 -0.289048 -0.670300
H 0.995846 -1.415303 -1.289984
H 2.239714 0.066730 1.530971
H 2.170958 1.346735 0.680643
H 0.420413 0.315976 2.577552
H 0.681919 -1.202821 2.462857
H -2.651453 1.371157 0.138751
H -2.853108 0.000878 0.826502
H 4.153696 0.006569 -0.470211
O 4.551442 -0.321795 -1.291912
H 3.778567 -0.615963 -1.790425

NO3-.HNO3.(H2O)8
H=-1173.121321
O -2.037420 -1.046826 -2.021168
O -0.180963 0.562914 -2.480661
O 1.699667 -0.841407 -1.133656
O -0.206814 2.370755 -0.321055
N -1.370179 2.134751 0.125858
O -2.346231 2.234922 -0.657757
O -1.541765 1.792842 1.311324
O 1.838415 0.955225 0.911286
O 4.597805 1.061341 0.704932
O -3.708456 -0.084642 -0.342080
O -0.580375 -2.463043 -0.440554
N -0.775301 -2.167475 0.797393
O 0.155935 -2.324906 1.598504
O -1.881627 -1.722980 1.155639
O 0.234360 0.187787 2.955091
H -0.241750 1.391326 -1.964212
H -1.546048 -1.706073 -1.414744
H -2.782209 -0.637835 -1.436569
H 0.605776 0.086560 -2.103720
H -1.314350 -0.307495 -2.264221
H 1.797650 -0.280303 -0.326271
H 1.183642 -1.617123 -0.853933
H 1.411207 0.645250 1.747255
H 1.200482 1.605818 0.550145
H -0.496815 0.686540 2.552927
H 0.057338 -0.745462 2.746483
H -3.447372 0.862117 -0.287531
H -3.304428 -0.510088 0.432789
H 3.627054 1.148514 0.814409
H 4.826727 0.284457 1.225204
H 4.688602 0.050670 -0.995630
O 4.595474 -0.663969 -1.647360
H 3.648821 -0.871906 -1.625019

NO3-.HNO3.(H2O)9
H=-1249.593180
O 0.967092 2.600206 1.376333
O -0.614401 2.910313 -0.505139
O -2.763771 1.727582 -0.018510
O 2.186482 -0.142266 -1.917270
N 1.060846 0.386362 -2.010409
O 0.015066 -0.313056 -1.924589
O 0.966884 1.627929 -2.184998

O -2.854370 -0.027390 -2.027805
O -2.996823 -2.262990 -0.417947
O -0.212243 -2.664071 -0.426431
O -2.191741 -0.442684 1.659471
N -1.019459 -0.308602 2.103086
O -0.200105 -1.259918 1.993716
O -0.682235 0.759079 2.648534
O 3.100359 1.312744 0.506547
O 2.674166 -1.041726 1.755139
H -0.164415 2.434910 -1.257898
H 0.558838 1.955354 1.985324
H 1.818324 2.179507 1.048627
H -1.534032 2.433524 -0.284293
H 0.061291 2.824622 0.326577
H -2.924649 1.113894 -0.790012
H -2.678037 1.130149 0.751365
H -3.005486 -0.883587 -1.562443
H -1.906018 -0.033388 -2.236971
H -0.083367 -1.889629 -1.009685
H -0.257832 -2.275743 0.470631
H 2.731280 -1.711055 1.037802
H 1.741086 -1.044730 2.027504
H -2.847806 -1.775405 0.411145
H -2.130643 -2.687174 -0.565198
H 3.030673 1.073413 -0.429493
H 3.040697 0.449961 0.994006
H 2.555804 -1.905385 -1.129625
O 2.607813 -2.653722 -0.511543
H 1.687197 -2.973784 -0.480169

NO3-.HNO3.(H2O)10
H=-1326.045178
O -0.845280 2.280837 -0.496058
O 3.026426 2.353373 -0.081058
O 1.282199 -0.268667 -2.308579
O 3.587576 -2.231913 1.035461
N 3.283314 -1.133459 0.596768
O 2.020325 -0.874455 0.352580
O 4.109210 -0.235125 0.360099
O -0.533041 -2.240685 -2.014244
O 0.038690 -2.575306 0.412154
O -1.217142 0.649270 3.122377
O -2.848915 2.247826 1.526857
O -3.528880 -0.154589 0.197499
N -3.062403 -0.451101 -0.939622
O -3.125951 -1.632754 -1.340142
O -2.522572 0.429888 -1.651538
O -1.779536 -1.630242 1.854907
O 1.377804 2.554473 -2.126412
H 1.684780 -0.388549 -1.429419
H -1.446149 1.630583 -0.919704
H -0.194904 2.542250 -1.176119
H 0.006264 -1.460296 -2.293003
H 1.211227 0.694819 -2.426185
H -0.229549 -2.493514 -0.603288
H -1.478289 -1.991997 -2.070444
H -0.722349 -2.178628 1.030776
H 0.885974 -2.043412 0.517352
H -2.185149 2.634715 0.934402
H -3.218189 1.513295 0.999851
H 2.078203 2.520855 -1.409001
H 1.673976 3.186019 -2.787322
H -0.364272 0.947729 2.748939
H -1.865014 1.268101 2.717183
H -1.540684 -0.824699 2.399546
H -2.563606 -1.354137 1.343368
H 3.486493 1.491633 -0.036813
H 2.362956 2.283643 0.635564
H 1.257025 0.586929 1.128994
O 0.884152 1.432612 1.446813
H 0.214345 1.690247 0.769569

NO3-.HNO3.(H2O)11
H=-1402.499747
O 2.392139 1.101514 2.167487
O -0.831106 2.849361 0.537851
O -0.085834 1.528886 2.534823
O -3.050720 -1.453689 -0.699569
N -3.787269 -0.877234 0.171826
O -4.586376 0.003173 -0.192508
O -3.692794 -1.213948 1.371220
O 1.061473 2.373529 -1.425750
O 3.427545 2.505283 0.099755
O 4.023354 -0.071537 -0.873592
N 3.356383 -1.046503 -0.485905
O 3.133117 -1.244871 0.735856
O 2.881388 -1.856243 -1.341830
O 1.412307 -3.641315 0.193828
O -0.504441 -1.741797 0.127352
O 0.613918 -0.222770 -1.788244
O -2.933849 2.201118 -0.966015
O -1.193613 -0.746144 2.530977
H -0.435497 2.082403 1.713744
H 2.658263 0.236035 1.799846
H 2.783320 1.753014 1.532857
H -0.589812 0.627140 2.578896
H 0.924505 1.340253 2.407776
H -2.153467 -0.824526 2.372479
H -0.815155 -1.182892 1.729615
H 0.019736 -2.578766 0.125208
H -1.435338 -1.895878 -0.158874
H 0.211586 -0.719330 -1.027697
H 1.402981 -0.744052 -2.018599
H 2.656680 2.612779 -0.488364
H 3.855332 1.695479 -0.232830
H 1.820913 -3.358112 1.021535

H 1.962171 -3.159115 -0.461802
H -3.656066 1.658243 -0.607955
H -2.580544 1.641482 -1.694410
H -1.681079 2.617767 0.071744
H -0.143310 2.796893 -0.157829
H 0.923002 1.395424 -1.562155
H 0.887124 2.791523 -2.274657
H -1.119252 0.040307 -2.613335
O -2.059781 0.260055 -2.720809
H -2.533713 -0.404748 -2.183307

NO3-.(HNO3)2
H=-842.465333
O -0.000027 0.331614 0.000052
N -0.000002 1.654809 0.000007
O 0.995305 2.223287 -0.440904
O -0.995299 2.223362 0.440835
O 2.938804 -0.276372 1.153831
N 3.146177 -0.787950 0.069444
O 2.132953 -0.802014 -0.841091
O 4.177058 -1.309736 -0.305414
O -2.938763 -0.276289 -1.153799
N -3.146181 -0.787868 -0.069416
O -4.176974 -1.309978 0.305245
O -2.133053 -0.801822 0.841201
H 1.329918 -0.284717 -0.442775
H -1.329921 -0.284630 0.442883

NO3-.(HNO3)2.(H2O)
H=-918.915450
O 0.223013 -2.230332 1.061369
O 4.499470 -0.479974 -0.640640
N 3.335604 -0.416656 -0.312252
O 2.866750 -0.728832 0.771103
O 2.486932 0.042090 -1.269872
O 0.123221 0.454660 -0.398580
N 0.021207 1.489947 0.348595
O 1.015430 2.092496 0.732901
O -1.143413 1.863954 0.672743
O -3.087931 0.785055 -0.596631
N -3.357128 -0.507294 -0.256566
O -4.272901 -1.015918 -0.865416
O -2.680571 -1.023578 0.615176
H 1.556767 0.187486 -0.834125
H -2.268287 1.099027 -0.053068
H -0.514286 -1.681581 0.766640
H 0.988014 -1.643892 1.004892

NO3-.(HNO3)2.(H2O)2
H=-995.381399
O -0.419050 -1.400585 1.437965
O -1.716055 0.956290 1.320171
O -3.760250 0.406546 0.092624
N -3.726169 -0.773375 -0.534575
O -2.716543 -1.470476 -0.426137
O -4.715256 -1.073112 -1.172959
O 3.391556 -0.066445 -0.468126
N 3.343820 -1.379922 -0.170097
O 4.348951 -2.012767 -0.411040
O 2.310400 -1.833409 0.313469
O -0.048282 2.744025 0.102726
N 1.041577 2.195650 -0.175029
O 1.176744 0.947672 0.137540
O 1.962754 2.795925 -0.721877
H -2.796365 0.609460 0.617566
H 0.462880 -1.281852 1.053660
H -0.977654 -1.725590 0.718100
H -1.130114 1.620723 0.879657
H -1.149906 0.156525 1.475011
H 2.456802 0.374954 -0.220964

NO3-.(HNO3)2.(H2O)3
H=-1071.837335
O 0.108654 -2.033517 0.608892
O -1.455906 0.002081 1.419649
O -3.867811 -0.332157 1.089012
N -4.293492 -0.287175 -0.183342
O -5.485785 -0.427946 -0.351305
O -3.465270 -0.105048 -1.074098
O -0.191830 2.259463 0.432815
N 0.971094 2.016376 0.052369
O 1.882048 2.831985 0.120195
O 1.204312 0.833145 -0.435948
O 3.631872 0.165819 -0.758007
N 3.934635 -0.887921 0.038557
O 3.058871 -1.336879 0.768230
O 5.070650 -1.294619 -0.047647
H -2.791683 -0.177102 1.139398
H 1.037812 -1.777720 0.697988
H -0.119819 -1.825210 -0.324434
H -1.034396 0.802602 1.035198
H -0.858748 -0.767635 1.220422
H 2.646306 0.457578 -0.573755
H -0.126143 -0.065598 -1.407265
O -0.671634 -0.809134 -1.724869
H -1.584363 -0.561425 -1.515988

NO3-.(HNO3)2.(H2O)4
H=-1148.294311
O 3.349376 -0.647861 1.143965
O 1.144938 0.112662 2.153529
O -0.847119 -1.401066 1.683239
O 0.804343 1.809038 0.306860
N 1.874508 2.338315 -0.228366
O 1.741935 3.112753 -1.162052

O 2.982122 2.028914 0.255270
O 0.975882 -2.479086 0.061390
N 1.415159 -2.034623 -1.034606
O 2.650517 -1.952108 -1.211096
O 0.618154 -1.667856 -1.935135
O -1.038683 0.218411 -0.920545
H 1.035806 0.881574 1.463884
H 3.236892 -1.279457 0.400447
H 3.488379 0.223291 0.727268
H 0.323983 -0.508398 2.035893
H 2.012050 -0.354649 1.842529
H -1.592902 -0.920310 1.289231
H -0.423759 -1.902827 0.949475
H -0.523860 -0.496587 -1.362193
H -0.415806 0.922540 -0.639785
H -2.454558 0.391649 -0.990529
O -3.494326 0.536839 -1.104222
N -4.115772 0.238111 0.059404
O -5.316059 0.386867 0.065017
O -3.434018 -0.151187 1.002375

NO3-.(HNO3)2.(H2O)5
H=-1224.752197
O -0.902250 1.739627 2.081137
O 1.598167 0.813978 2.276910
O 2.946316 1.351173 0.252456
O 1.591049 -1.846831 1.426721
N 0.525296 -1.938461 0.753271
O -0.496475 -1.312158 1.161661
O 0.484379 -2.613693 -0.284384
O 3.490867 -1.034931 -0.489681
O 1.208379 1.586312 -1.641311
N 0.064964 1.874759 -1.106767
O -0.926565 1.215698 -1.410516
O 0.032441 2.814146 -0.282143
O 1.641299 -1.035268 -2.465773
H 1.515650 -0.160367 2.231995
H -0.815681 2.268593 1.264081
H -1.495594 1.015716 1.833741
H 2.363704 1.203042 1.139048
H 0.661677 1.161722 2.273300
H 3.270019 0.409640 -0.033504
H 2.311025 1.633707 -0.489683
H 2.930661 -1.120347 -1.305882
H 3.025452 -1.582374 0.171470
H 0.979055 -1.605172 -2.048379
H 1.309575 -0.121460 -2.344222
O -2.507004 -1.393219 -0.517285
N -3.389450 -0.419100 -0.154628
O -4.347800 -0.299417 -0.877754
O -3.154709 0.218005 0.861308
H -1.725963 -1.350470 0.134154

NO3-.(HNO3)2.(H2O)6
H=-1301.210922
O -0.804083 2.530570 -1.757352
O -2.905702 1.402292 -0.887095
O -2.385893 1.380378 1.543683
O -2.158394 -0.914091 -1.812560
N -2.098295 -1.769842 -0.851903
O -3.078565 -1.876350 -0.092368
O -1.068126 -2.444666 -0.716937
O 0.054969 2.595954 0.944383
N 1.043575 1.819767 0.927372
O 1.888297 1.950922 -0.013520
O 1.191914 0.948614 1.795018
O -2.247767 -1.094674 2.492094
O 0.325254 0.191092 -2.451742
O 0.301276 -1.823974 1.809437
H -2.812661 0.468447 -1.259132
H -0.336956 2.845380 -0.962426
H -0.267909 1.756520 -2.077225
H -2.746135 1.359970 0.173024
H -2.094933 1.928918 -1.279584
H -2.339167 0.476718 1.975630
H -1.500100 1.788277 1.613859
H -1.305583 -1.373022 2.411045
H -2.681745 -1.577510 1.767632
H 0.112590 -2.167697 0.922143
H 0.793414 -1.001056 1.670235
H -0.445093 -0.399113 -2.328152
H 1.008651 -0.148824 -1.857616
H 3.013554 0.909715 0.025242
O 3.838500 0.288865 0.008579
N 3.398140 -0.967318 -0.276887
O 4.239849 -1.829952 -0.253631
O 2.208240 -1.110601 -0.531080

NO3-.(HNO3)2.(H2O)7
H=-1377.660946
O -2.712010 1.069726 0.889581
O -2.937009 -1.161411 -0.596813
O -1.686735 -2.723392 0.921954
O 0.322826 -3.510169 -0.298583
O -0.128820 -1.970796 -2.513988
N -0.061454 -0.760726 -2.198838
O -1.076015 -0.022942 -2.374621
O 0.977948 -0.271624 -1.704833
O -0.980117 2.266552 -0.829140
O 2.560126 -2.048079 -0.165595
O -1.150114 -0.677740 2.537498
N 0.048569 -0.373385 2.251444
O 0.910566 -1.255080 2.158931
O 0.324634 0.842440 2.048672
O 2.662518 0.908461 0.909794
N 2.767117 1.910901 -0.017873

O 1.839452 2.701099 -0.103009
O 3.792496 1.920180 -0.646952
H -2.460509 -0.918503 -1.414968
H -2.260102 0.713206 1.672765
H -2.008559 1.542242 0.383687
H -2.227934 -2.124732 0.241153
H -2.987768 -0.312219 -0.077617
H -0.786243 -3.078283 0.452409
H -1.457610 -2.117985 1.679957
H 1.188774 -3.087943 -0.050694
H 0.189125 -3.169065 -1.208983
H 2.214117 -1.439281 -0.846469
H 2.567881 -1.507446 0.635375
H -0.910373 1.546397 -1.491528
H -0.077028 2.430991 -0.523003
H 1.740861 0.973357 1.345299
H -3.642787 3.033628 0.293505
O -3.442539 3.755556 -0.315674
H -2.579126 3.495863 -0.671810

NO3-.(HNO3)2.(H2O)8
H=-1454.125530
O -4.525805 -1.107883 -0.781276
O -3.262666 0.527137 -2.158344
O -1.157459 -0.846762 -2.552001
O -4.543150 -0.458617 1.830469
O -1.907607 2.128924 -0.602331
N -2.319538 2.008369 0.612230
O -1.494866 1.943379 1.534738
O -3.545243 1.960266 0.811735
O -2.202112 -2.534063 -0.522836
N -1.494739 -2.140465 0.457194
O -2.029548 -1.791199 1.527745
O -0.251782 -2.094788 0.318950
O 0.505085 0.932686 -1.254129
O 0.766683 0.197008 1.394598
O 3.330228 1.189042 1.271746
O 3.035552 2.078773 -1.283663
H -2.906778 1.275500 -1.585233
H -3.834257 -1.796281 -0.648687
H -4.734046 -0.812066 0.143847
H -2.421742 -0.032760 -2.406528
H -3.880368 -0.114013 -1.556962
H -0.420330 -0.328383 -2.151427
H -1.267174 -1.641223 -1.995030
H 0.706062 0.555008 -0.368624
H -0.235201 1.543997 -1.054478
H 0.007532 0.754021 1.643211
H 0.432332 -0.720459 1.305022
H -4.232188 0.462698 1.786602
H -3.730643 -0.984692 1.934099
H 3.223369 1.650090 0.402618
H 2.454109 0.792268 1.465564
H 3.641160 1.409642 -1.626956
H 2.132277 1.745803 -1.468772
H 4.429366 0.273751 1.193928
O 5.269020 -0.382072 1.235915
N 5.681547 -0.660513 -0.020332
O 6.625523 -1.412810 -0.109153
O 5.083850 -0.139601 -0.956642

NO3-.(HNO3)2.(H2O)9
H=-1530.586928
O -1.874130 3.329451 -0.398172
O 0.608299 3.235079 -0.626304
O 0.631694 1.319247 -2.305438
O 0.259697 -1.192658 1.592920
N -0.695082 -0.510422 2.062044
O -0.520575 0.730133 2.261143
O -1.781620 -1.030686 2.330717
O 1.667292 2.032753 1.360765
O -2.942042 2.067742 1.714194
O 1.615161 -0.756882 -0.766745
O -2.105394 1.070205 -1.998671
N -2.701326 0.099831 -1.436836
O -3.539887 0.314903 -0.545628
O -2.440916 -1.074155 -1.796069
O -3.229449 -2.787357 0.417831
O -0.631038 -3.711182 0.418397
O 0.059560 -2.483950 -1.877486
H 1.061201 2.811437 0.192141
H -2.151273 2.630319 -1.029900
H -2.257189 3.021082 0.466136
H 0.673717 2.523366 -1.371276
H -0.434187 3.342935 -0.451510
H 1.043899 0.574936 -1.814373
H -0.312479 1.075684 -2.402945
H 1.079731 -1.481834 -1.244779
H 1.238914 -0.735710 0.139458
H -3.046955 -2.164283 1.136095
H -3.208261 -2.230946 -0.380104
H -2.200440 1.593279 2.134329
H -3.358637 1.400081 1.145984
H -0.301236 -3.016033 1.012603
H -1.604486 -3.573318 0.429198
H -0.187651 -3.070280 -1.113548
H -0.755800 -1.993904 -2.080858
H 0.976726 1.458107 1.767552
H 2.445329 1.481227 1.194976
O 4.018993 0.365731 0.859633
N 4.731130 -0.235210 0.059920
O 5.937165 -0.253663 0.054500
O 4.125635 -0.957117 -0.911785
H 3.088454 -0.866265 -0.795485

NO3-.(HNO3)2.(H2O)10

H=-1607.040922
O -2.200753 -2.453342 1.336759
O -0.182462 -2.877760 -0.016358
O 1.895011 -2.247618 1.511797
O 0.743659 0.122377 -1.018992
N -0.381151 0.155211 -1.585594
O -0.940141 1.251206 -1.816497
O -0.931138 -0.924758 -1.905678
O -4.339819 -2.069377 0.053950
O 3.157212 1.676722 -1.032464
O 5.287879 0.447099 -0.719547
N 5.058637 -0.801499 -0.277385
O 6.029808 -1.449821 0.038118
O 3.888594 -1.182974 -0.242276
O -1.855714 0.042035 2.117867
N -2.654213 0.917470 1.632203
O -2.334788 2.109521 1.646780
O -3.748908 0.542636 1.151638
O -3.829427 1.469804 -1.608116
O -0.274699 3.348101 0.030952
O 2.169943 2.835080 1.135602
O -3.768926 -1.169422 -2.393383
H -0.192847 -2.272562 -0.783273
H -2.105830 -1.524837 1.707857
H -3.066920 -2.426553 0.761938
H 0.651249 -2.695983 0.506192
H -1.324141 -2.671397 0.742269
H 2.711428 -2.115914 1.005651
H 1.635723 -1.348049 1.800955
H 2.873889 2.184832 -0.215752
H 2.387503 1.122800 -1.250034
H -2.868357 1.538477 -1.735011
H -3.949469 1.423748 -0.645773
H -4.176670 -1.849072 -0.906579
H -4.488268 -1.203223 0.467950
H -0.367716 2.731775 -0.715497
H -0.971723 3.078615 0.648505
H 1.316005 3.172021 0.776503
H 1.892949 2.063194 1.659812
H -3.980093 -0.220657 -2.245386
H -2.797873 -1.163190 -2.433730
H 4.336303 0.934492 -0.858171
O 0.920889 0.355101 1.781593
H 0.832384 0.351085 0.810170
H -0.000189 0.395225 2.102862

NO3-.(HNO3)2.(H2O)11
H=-1683.495360
O 0.911940 2.448915 1.911153
O -1.273180 1.337768 2.309396
O -1.088961 -1.102137 1.661592
O 3.341024 -2.305876 -2.043354
O 3.278300 -3.517132 0.474631
O -2.232513 0.920862 -1.702040
N -1.214661 1.658347 -1.627872
O -1.368197 2.861928 -1.277919
O -0.074876 1.223598 -1.880536
O 1.092692 3.861703 -0.337224
O -1.982608 -1.628803 -0.949545
O -4.304266 -2.546437 -0.382938
N -4.892310 -1.521386 0.306659
O -4.216376 -0.525053 0.518469
O -6.033763 -1.713120 0.641188
O 2.913315 0.707460 1.062217
N 3.497787 0.948244 -0.053505
O 3.322269 2.018025 -0.635865
O 4.259825 0.065502 -0.523982
O 0.622097 -1.529855 -1.695812
O 4.983551 -1.600371 1.618143
O -3.157349 2.326519 0.807466
H -2.018782 1.759263 1.765422
H 1.654902 1.847858 1.684931
H 0.910580 3.104360 1.159896
H -1.208586 0.330978 2.063750
H -0.336442 1.822666 2.132095
H -1.533089 -1.297803 0.811578
H -0.154151 -1.385222 1.524629
H -1.124637 -1.925496 -1.327668
H -2.091088 -0.703217 -1.285292
H 0.440985 -0.596418 -1.918671
H 0.916385 -1.515832 -0.752922
H 0.306527 3.578724 -0.841061
H 1.823054 3.307691 -0.657503
H 4.473031 -1.160377 2.306876
H 4.844938 -1.019500 0.842711
H 4.011435 -3.061691 0.933859
H 3.385654 -3.267160 -0.469448
H -2.717242 2.680443 0.007444
H -3.693280 1.586018 0.490894
H -3.358987 -2.233761 -0.629338
H 2.394001 -2.109321 -2.169844
H 3.725770 -1.457613 -1.774235
O 1.448216 -1.628683 0.905432
H 2.048248 -2.424412 0.851796
H 2.035935 -0.855128 1.029328

O-
H=-75.131976
o 0.000000 0.000000 0.000000

O-.(H2O)
H=-151.624085
o 0.049978 1.338225 0.000000
h 0.102046 -0.059435 0.000000
o 0.049978 -1.166780 0.000000
h -0.901697 -1.312125 0.000000

O-.(H2O)2
H=-228.092908
O -1.690309 -0.000116 0.000000
O 0.838776 -1.184078 -0.064554
H -1.028387 -0.722019 -0.052974
H -1.028257 0.721696 0.052954
H 1.080525 0.816996 -0.795606
H 1.080701 -0.816587 0.795528
O 0.838460 1.184183 0.064566

O-.(H2O)3
H=-304.561358
O -0.421535 -0.387509 0.041929
H 1.135859 -1.092037 0.100432
O 2.123405 -1.248424 0.011762
H 2.363994 -0.529001 -0.582469
H 0.536168 0.991143 -0.078303
O 1.279842 1.671565 -0.036388
H 1.881853 1.244798 0.582912
H -2.084285 -0.354353 0.024947
O -3.074488 -0.166405 -0.005583
H -3.091378 0.785636 -0.141281

O-.(H2O)4
H=-381.026125
O 0.000093 0.000658 -1.394776
H 1.104413 -1.111152 -0.512659
O 1.541156 -1.414506 0.323101
H 1.751474 -0.555050 0.720767
H 1.110482 1.104032 -0.513305
O 1.415336 1.539998 0.322386
H 0.556658 1.750858 0.721278
H -1.104234 1.109856 -0.512091
O -1.541304 1.414233 0.323230
H -1.752556 0.555265 0.721362
H -1.110135 -1.103413 -0.513234
O -1.415208 -1.540277 0.321910
H -0.556690 -1.751252 0.721080

O-.(H2O)5
H=-457.487579
O -0.244821 -0.403175 -1.415406
H -1.689496 -0.831406 -0.531166
O -2.332725 -0.922148 0.224186
H -1.777346 -1.299261 0.916788
O -1.296553 1.781603 -0.328707
H -1.956339 1.203633 0.085398
H -0.900588 1.156379 -0.991340
O 0.695171 -1.725415 0.666773
H 0.301765 -1.431043 -0.205262
H 1.626674 -1.506492 0.519158
O 2.422236 0.217638 -0.751157
H 1.580878 0.016073 -1.219598
H 2.108729 0.738844 0.007881
H 0.068147 1.487481 0.920433
O 0.761591 1.092783 1.489208
H 0.598396 0.135504 1.418535

O-.(H2O)6
H=-533.951105
O -1.585537 1.814750 -0.286301
O 0.139328 1.079446 1.814839
O 2.199729 1.090065 -0.153891
O 0.218711 0.284595 -1.604561
O -2.178985 -0.928318 -0.708348
O -0.499398 -1.643707 1.440652
O 1.543613 -1.815005 -0.528956
H -0.908484 1.512117 -0.938972
H -2.151946 1.020796 -0.262092
H -0.530827 1.439858 1.194504
H 0.958051 1.090590 1.282677
H 1.462699 0.845414 -0.822831
H 2.435577 2.002290 -0.344526
H -1.424097 -0.671254 -1.275656
H -1.735681 -1.284422 0.091157
H 0.238784 -1.866830 0.839178
H -0.302126 -0.722509 1.714769
H 2.236569 -1.242049 -0.177444
H 1.021785 -1.178604 -1.088233

O-.(H2O)7
H=-610.413172
O 1.571294 -1.751992 0.569579
O 0.428221 0.025210 2.377889
O -0.916714 -2.041541 -0.745222
O -0.353193 0.032018 -2.103294
O -1.541575 1.701444 -0.606383
O 2.154470 0.330595 -1.203482
O 0.989804 2.122839 0.607453
O -2.122967 -0.387110 1.186958
H 0.741661 -2.027698 0.136054
H 1.260367 -1.190433 1.316482
H -0.502807 -0.128858 2.117046
H 0.679733 0.835847 1.883027
H 0.093691 2.143288 0.222119
H 1.506428 1.591671 -0.039085
H 2.077700 -0.452079 -0.611622
H 1.392905 0.240636 -1.817224
H -2.097882 0.415410 0.631248
H -1.840179 -1.094695 0.576353
H -2.002758 2.380078 -1.105885
H -1.105656 1.054898 -1.298360
H -0.713488 -1.236453 -1.375232
H -1.164437 -2.783322 -1.302916

O-.(H2O)8
H=-686.868507
H 0.406933 1.002683 -1.616147
O 1.317949 1.444389 -1.521417
H 1.416445 2.035611 -2.272299
O 0.747740 2.232253 1.147032
H -0.223813 2.246685 1.035081
H 1.073134 2.100653 0.237372
O -1.940654 2.051219 0.312682
H -2.527995 1.424462 0.751879
H -1.632712 1.537082 -0.476931
O -1.007092 0.269226 -1.519487
O 0.304220 -2.157666 -1.197879
H -0.175630 -1.359519 -1.510637
H 1.215524 -1.829927 -1.025458
O -0.650603 -1.939087 1.394948
H -0.294276 -2.163611 0.503859
H -1.443063 -1.418505 1.182627
O 1.570642 -0.321802 1.995926
H 1.268668 0.596606 1.834320
H 0.765347 -0.875662 1.919948
O 2.660749 -0.845236 -0.493615
H 2.401306 -0.016832 -0.933148
H 2.397975 -0.700145 0.446483
H -2.270736 -0.442492 -0.693884
O -2.913285 -0.788219 -0.008062
H -3.094433 -1.697711 -0.264093

O-.(H2O)9
H=-763.328100
O 0.889170 -1.300129 -1.056261
O -0.136001 -1.906720 1.352175
O -1.700424 -0.850680 -1.759986
O -2.805199 -1.365831 0.530526
O -2.440257 1.357061 1.259479
O 0.299791 0.794379 1.647002
O 1.075145 1.348634 -1.079781
O 3.113484 1.277757 0.871179
O 3.449398 -1.193702 -0.355899
H 3.874184 -0.980135 -1.192435
H 2.479474 -1.319863 -0.597106
H 3.337220 0.370388 0.568115
H 2.364199 1.156386 1.477094
H 1.904888 1.526711 -0.593928
H 1.082823 0.372949 -1.278565
H 0.200097 -0.188488 1.666110
H 0.400808 1.004773 0.699278
H -1.530206 1.264040 1.606523
H -2.728250 0.436650 1.117809
H -2.440603 -1.207221 -0.402110
H -3.579049 -1.927276 0.436657
H -1.095656 -1.886489 1.187144
H -0.742193 -1.066811 -1.602339
H 0.271458 -1.950696 0.451891
H -1.737568 0.132756 -1.802079
O -1.665820 1.897729 -1.401771
H -0.695685 1.945080 -1.314669
H -2.000240 1.849276 -0.480704

O-.(H2O)10
H=-839.784692
O 1.782146 2.412538 -0.510784
O -0.622549 1.346296 -0.704146
O -0.060639 -0.859297 -1.827858
O 2.587497 -0.095623 -1.715382
O -3.233746 2.060653 -0.382178
O 0.576023 -2.275215 0.377646
O -0.588322 -0.117578 1.593060
O -2.177389 -2.211253 -0.766064
O -3.260714 -0.425957 1.050770
O 3.077201 -1.226987 0.783265
O 1.946881 1.069794 2.027976
H -2.301692 1.854633 -0.639573
H -3.149823 2.741633 0.292448
H 0.836889 2.115837 -0.650967
H -0.602795 0.883083 0.172510
H 2.484741 0.833647 -1.455879
H 1.662077 -0.410580 -1.875056
H 1.970502 1.641049 1.240441
H 1.026909 0.751742 2.077096
H -1.566889 -0.237757 1.622134
H -0.219357 -0.960973 1.227447
H -1.623292 -2.764281 -0.200857
H -1.502763 -1.698433 -1.287765
H -0.480639 0.564352 -1.351320
H 1.516278 -2.078750 0.606326
H 0.484596 -1.972707 -0.555398
H 3.064258 -0.868332 -0.132269
H 2.812084 -0.463100 1.334143
H -3.429431 0.396902 0.555276
H -3.016822 -1.088224 0.363980
H 1.824067 3.341305 -0.753162

O-.(H2O)11
H=-916.243272
O -3.091626 0.160483 1.739652
O -0.490257 -0.553423 1.558858
O -1.753988 0.131463 -1.787559
O -3.486919 -1.503382 -0.477831
O 0.563879 1.500446 -1.937912
O 2.929850 0.350665 -1.502149
O 3.112273 1.276107 0.936159
O 2.140031 -1.166954 1.899113
O 2.248888 -2.203246 -0.545591
O -2.396034 2.151718 -0.082300
O 0.366049 1.965580 0.841697

O -0.588192 -1.987302 -0.766182
H -2.952940 0.972601 1.201843
H -3.400855 -0.487394 1.070806
H 2.667021 -3.068417 -0.543842
H 2.229248 -1.889057 0.405175
H 2.772015 -0.601235 -1.365565
H 2.055839 0.727634 -1.803536
H 0.363271 -2.102185 -0.926337
H -0.929896 -1.254510 -1.358196
H 1.181868 -0.980620 2.015662
H 2.553245 -0.293905 1.752255
H 0.467858 1.827759 -1.022232
H -0.273331 0.976629 -2.064129
H 0.061137 1.065705 1.106679
H 1.324714 1.961693 1.004273
H -2.828325 -2.202963 -0.380995
H -3.003913 -0.851959 -1.054638
H -2.257684 1.526099 -0.838548
H -1.503032 2.413278 0.186249
H -1.423149 -0.336561 1.818936
H -0.575710 -1.100165 0.739016
H 3.930524 1.760369 1.073724
H 3.110459 0.959958 -0.024232

OH-
H=-75.798020
O 0.000000 0.000000 0.107877
H 0.000000 0.000000 -0.863017

OH-.(H2O)
H=-152.293704
O -1.225424 -0.093734 -0.059448
H -1.462830 0.629999 0.530475
H -0.025390 -0.008900 -0.096821
O 1.226288 0.092303 -0.063053
H 1.481308 -0.609646 0.546349

OH-.(H2O)2
H=-228.767581
O 0.018079 1.384348 -0.112013
H -0.078692 1.842123 0.730227
H 1.017401 0.217775 0.047775
O 1.533536 -0.686863 0.063868
H 1.061883 -1.170250 -0.622554
H -1.062187 0.208475 -0.112346
O -1.557554 -0.687105 -0.016497
H -0.890892 -1.181165 0.474034

OH-.(H2O)3
H=-305.238511
O -0.003901 0.001235 1.427088
H -0.011086 0.006820 2.385694
H 1.244298 -0.339393 0.375338
O 1.676829 -0.565596 -0.503415
H 0.948762 -1.057452 -0.908444
H -0.917327 -0.906037 0.371180
O -1.325547 -1.168508 -0.509523
H -1.385581 -0.292439 -0.915612
O -0.346965 1.732086 -0.509846
H -0.327695 1.244952 0.369880
H 0.445298 1.349811 -0.912473

OH-.(H2O)4
H=-381.705290
O 0.000020 -1.528528 -0.490442
O -0.000229 -0.164131 1.836021
O 2.081834 -0.030598 -0.533231
O -2.081791 -0.030496 -0.533437
H 0.000074 -2.480225 -0.610575
H 1.326451 -0.709933 -0.647190
H 2.144261 0.012750 0.428666
H -1.326476 -0.709854 -0.647432
H -2.144046 0.012956 0.428469
H -0.000158 -0.819259 1.086086
H -0.000084 0.685578 1.366054
O 0.000129 1.913481 -0.313574
H 0.771229 1.365045 -0.563282
H -0.770950 1.365124 -0.563491

OH-.(H2O)5
H=-458.168037
O -0.319112 -0.631820 1.307323
O 0.243627 -1.380458 -1.281912
O 1.263199 1.332342 -1.141591
O -0.936994 1.825221 0.536860
O -2.504629 -0.545376 -0.346858
H -0.496260 -1.067279 2.144768
H 0.021675 -1.325959 -0.323355
H 0.551331 -0.473680 -1.471699
H 1.803383 0.904976 -0.449235
H 0.486224 1.661824 -0.640108
H -0.657391 0.994901 1.020562
H -1.692235 1.484354 0.035460
H -1.977055 -0.848338 -1.099091
H -1.842944 -0.643300 0.385229
H 1.326105 -0.503507 1.140114
O 2.281386 -0.480837 0.819429
H 2.257356 -1.136563 0.111341

OH-.(H2O)6
H=-534.629588
O -2.227794 0.492702 -0.835302
O 0.356242 -0.366316 -1.595157
O -1.161192 -2.072016 -0.152103
O 0.238167 -0.868585 1.965082
O 1.142853 1.992851 -0.595415

O 2.312606 -0.704968 0.028497
O -0.873156 1.664677 1.371766
H -1.367265 0.292832 -1.263642
H -1.952998 0.976318 -0.030198
H -0.177121 1.949441 0.745750
H -0.533052 0.810783 1.713736
H 1.054765 -0.750232 1.441578
H -0.325058 -1.393866 1.355701
H -1.904016 -1.445282 -0.199031
H -0.527955 -1.613511 -0.765728
H 1.916283 1.605884 -0.167385
H 0.777089 1.209395 -1.095022
H 2.642860 -1.606595 -0.014305
H 1.577829 -0.640401 -0.687713
H 0.516827 -0.501511 -2.532687

OH-.(H2O)7
H=-611.091967
O 0.000082 1.025976 -1.474615
H 0.000151 1.544091 -2.284142
O -2.455071 1.139845 -0.329680
H -1.622141 1.101597 -0.873538
H -2.137335 1.582593 0.470109
O -0.000707 -1.494530 -1.563399
H -0.000408 -0.451029 -1.615312
H -0.000463 -1.823862 -2.465418
O 2.455501 1.138340 -0.330141
H 1.622348 1.100637 -0.873730
H 2.138473 1.581451 0.469723
O 0.001537 1.936029 1.222355
H 0.000415 1.766980 0.259731
H 0.000662 1.033223 1.616918
O 2.057740 -1.554554 0.348142
H 2.379568 -0.650908 0.145052
H 1.409519 -1.717081 -0.364433
O -0.000131 -0.706970 2.091937
H 0.772847 -1.074705 1.614138
H -0.773511 -1.073915 1.614149
O -2.058826 -1.553298 0.348583
H -2.380159 -0.649467 0.145516
H -1.410962 -1.716302 -0.364204

OH-.(H2O)8
H=-687.547859
O -1.027785 -1.595726 -1.444712
O 1.277495 -0.479180 -1.463815
O -0.813632 -2.070089 1.318778
O -0.137222 1.895223 -1.348563
O 1.941756 -1.842877 0.773696
O 2.762081 0.992238 0.191734
O 0.317452 2.086148 1.356974
O -1.941232 0.458814 1.793982
O -2.609927 0.673438 -0.898361
H -2.241361 -0.179662 -1.191173
H -2.510264 0.641690 0.081689
H 0.368042 1.061034 -1.495617
H -1.073778 1.601169 -1.300662
H 2.652588 1.917147 -0.051381
H 2.227036 0.485775 -0.486462
H -0.095601 -1.160655 -1.503371
H 1.768915 -0.635290 -2.275343
H 0.121495 2.148764 0.390469
H 1.112818 1.532503 1.365422
H -1.164501 1.055284 1.783285
H -1.560231 -0.444601 1.764981
H 1.725436 -1.477590 -0.124871
H -1.015115 -2.026873 0.364084
H 0.164387 -2.036580 1.328939
H -1.037902 -2.321372 -2.073792
H 2.406154 -1.104650 1.186114

OH-.(H2O)9
H=-764.006711
O 1.516045 -1.870039 1.054726
O 2.340311 0.447961 1.024526
O 0.400064 2.229191 1.449052
O -1.932578 0.802011 1.809133
O -1.196257 -1.890062 1.510785
O -1.811978 -1.607147 -1.275916
O -2.364110 0.913800 -0.860908
O -0.046748 2.332451 -1.431322
O 1.924877 0.664665 -1.554445
O 0.976746 -1.969761 -1.652427
H 1.926311 -0.862658 1.105635
H 2.101968 -2.456883 1.538725
H 1.155435 1.578118 1.385200
H 3.210447 0.631998 1.386966
H -1.087568 1.311822 1.817314
H 0.237267 2.479528 0.525332
H -1.668099 -0.142378 1.851320
H -1.484946 -1.946482 0.583237
H -0.214306 -1.901774 1.452659
H 1.225688 -2.070560 -0.706852
H 0.005564 -1.890380 -1.634244
H 2.198387 0.616079 -0.584107
H 1.621744 -0.247187 -1.755974
H 0.717513 1.667428 -1.533276
H 0.110509 3.029627 -2.072875
H -2.321456 0.941638 0.133012
H -1.608667 1.465366 -1.151528
H -2.030254 -0.626419 -1.173104
H -2.546514 -2.001437 -1.753066

OH-.(H2O)10
H=-840.466155
O -2.757824 1.409235 -0.633138

O -2.840321 0.165862 1.668534
O -1.991064 -0.820202 -1.942942
O -2.104334 -2.341962 0.447887
O 0.609024 -1.634543 0.446803
O 3.245778 -2.170127 0.674655
O 0.691991 -0.242394 -1.753561
O -0.062388 2.041189 -0.753476
O 3.104652 0.396895 -0.632359
O 2.229418 2.531937 0.810507
O -0.097787 0.737431 1.701924
H -2.597475 0.626577 -1.225724
H -2.125554 -1.479266 -1.232442
H -2.438737 -1.595975 0.975551
H -2.878120 0.684737 0.802682
H 0.254604 1.232944 -1.267971
H 0.648305 -1.226343 -0.475549
H 0.448026 -0.851834 1.020418
H -0.183414 1.210234 0.842257
H 2.668886 1.781945 0.337163
H 3.330086 -0.401077 -0.121759
H 1.728030 2.100657 1.515247
H -1.901470 1.881042 -0.666792
H -1.128827 -2.265306 0.511618
H 0.955199 -0.448079 -2.654347
H -1.018766 0.568273 1.970852
H 0.737736 2.487408 -0.415451
H 2.258794 -2.065389 0.690713
H 3.408941 -2.776075 -0.054588
H 2.250900 0.167612 -1.084790
H -1.017142 -0.633254 -1.921922
H -3.617160 0.414595 2.176161

OH-.(H2O)11
H=-916.921055
O -2.834026 1.944846 -0.076475
O -2.750858 0.108108 1.798389
O -0.001375 2.019153 -0.208124
O 0.076955 -0.191728 -1.696991
O 0.114889 -1.728844 0.233825
O -0.032100 0.158236 1.959533
O -3.017560 -1.984942 -0.041259
O -2.581680 -0.169558 -1.977190
O 2.915271 -0.024522 -1.950784
O 2.795043 1.829137 -0.047329
O 2.802714 0.043556 2.009745
O 2.746149 -2.030840 0.174653
H 0.023410 1.234662 -0.824947
H -0.937012 2.279710 -0.174275
H -1.789424 0.106387 2.040747
H -2.881457 -0.719790 1.291383
H 0.018806 -0.654260 1.328799
H -0.636741 -2.330556 0.236361
H -2.751173 0.654836 -1.486352
H -1.598712 -0.190901 -2.086095
H -2.845511 1.278801 0.681350
H -3.535579 2.576880 0.100850
H 0.038334 0.911539 1.341358
H 0.089038 -0.914127 -0.888149
H -2.841919 -1.371655 -0.807216
H -3.854917 -2.412310 -0.241719
H 1.750180 -2.095011 0.209910
H 2.914318 -1.515289 -0.630562
H 2.885984 -0.764820 1.449759
H 2.866866 1.264132 0.765697
H 1.874339 2.147895 -0.048759
H 2.895197 0.707789 -1.267264
H 0.951726 -0.221478 -2.114093
H 1.852375 0.064824 2.240520
H 3.644490 0.181922 -2.541254

OH
H=-75.754480
O 0.000000 0.000000 0.108539
H 0.000000 0.000000 -0.868313

HNO3
H=-280.965339
O -0.261667 1.232760 0.000024
N -0.155836 0.028271 -0.000114
O 1.175793 -0.476686 -0.000013
O -0.992978 -0.821916 0.000054
H 1.721669 0.328832 0.000279

NO3-.OH
H=-356.233865
O -0.079625 -0.956067 -0.003040
N 0.753650 0.009164 -0.000504
O 1.977333 -0.230690 0.003023
O 0.319717 1.186656 -0.001737
H -1.693340 -0.218250 0.000210
O -2.665201 0.019364 0.002169

NO3-.OH.(H2O)
H=-432.692277
O 2.715692 -1.183939 0.000311
O -0.183123 -0.548153 -0.000291
N -0.032051 0.719472 -0.000068
O -1.036025 1.454449 0.000547
O 1.126589 1.202064 -0.000485
O -2.886788 -1.216889 -0.000057
H 2.451359 -0.246313 0.000116
H 1.833994 -1.579790 0.000111
H -1.951755 -0.870457 0.000049

NO3-.OH.(H2O)2
H=-509.148522

O -3.297375 -0.404167 0.364098
O -0.601158 -1.374685 -0.265486
N 0.474683 -0.765134 -0.103411
O 0.470382 0.516376 -0.033558
O 1.555163 -1.373665 -0.013314
O 3.318091 1.002926 0.210488
H -2.454945 -0.858996 0.202090
H -3.058067 0.518205 0.189435
H 2.367915 1.194336 0.144620
H 3.277027 0.037805 0.179456
O -1.756630 2.016179 -0.237624
H -0.962490 1.400880 -0.188554

NO3-.OH.(H2O)3
H=-585.606991
O 1.833343 -1.429705 -1.336605
O -0.526489 -0.390632 -0.017557
N -0.929805 0.813937 -0.018422
O -2.158473 1.045428 0.003506
O -0.108290 1.756239 -0.040701
O 2.645525 1.070212 -0.138559
O 1.880084 -1.093621 1.533270
H 2.061750 -1.699180 -0.430178
H 0.934839 -1.081934 -1.203132
H 2.320157 -0.283211 1.210533
H 0.954908 -0.931438 1.285766
H 1.725605 1.398498 -0.096458
H 2.589520 0.356959 -0.798093
H -3.054643 -0.560462 0.013011
O -3.693638 -1.320019 0.015085

NO3-.OH.(H2O)4
H=-662.062566
O 2.402127 -0.035108 -1.266251
O 2.155964 -1.282373 1.307703
O 0.124364 0.749164 1.309753
N -0.896482 0.498357 0.607895
O -1.334881 -0.686710 0.557420
O -1.459960 1.402739 -0.032724
O 1.245264 2.501766 -0.569456
O 0.652905 -2.251606 -0.913308
H 2.058581 0.873551 -1.177487
H 2.589264 -0.316854 -0.351019
H 1.718893 -1.917916 0.710762
H 1.433730 -0.667085 1.526822
H 0.366765 2.569911 -0.964941
H 1.024582 2.093743 0.289685
H -0.132726 -1.849638 -0.508459
H 1.141018 -1.497885 -1.298890
H -2.991307 -0.595129 -0.285927
O -3.902463 -0.670523 -0.667614

NO3-.OH.(H2O)5
H=-738.521914
O 1.462947 -0.937599 1.797173
O 2.874514 1.046262 0.398278
O 1.092181 -2.743348 -0.345979
O -0.738413 -0.013902 0.228966
N -1.799881 -0.679179 -0.032885
O -2.890631 -0.071320 -0.059170
O -1.725076 -1.898818 -0.257659
O 0.764021 2.695835 -0.655159
O 1.394660 -0.172448 -1.624917
H 1.527663 -1.700967 1.187547
H 0.598721 -0.571209 1.547021
H 0.612876 -0.147590 -1.042043
H 2.105251 0.185733 -1.049085
H 1.382735 -2.073454 -0.990393
H 0.130172 -2.598031 -0.297416
H 2.226875 1.769873 0.319529
H 2.487067 0.408189 1.034464
H 0.787907 1.980158 -1.305412
H -0.131541 2.653717 -0.272009
H -2.296132 1.660434 0.337676
O -1.838255 2.493763 0.613507

NO3-.OH.(H2O)6
H=-814.982405
O 1.359476 -1.630386 1.706086
O -1.187383 -0.612749 2.021279
O -1.246074 1.514865 0.402500
O 3.038917 0.473410 1.001581
O 1.369816 2.586496 0.159423
O -0.376254 -0.201068 -1.601203
N 0.832020 -0.566154 -1.458904
O 1.096314 -1.754538 -1.206381
O 1.746119 0.289196 -1.571070
O -2.505590 -1.559728 -0.272810
H 2.520890 1.303281 0.991606
H 2.992628 0.206267 0.069428
H 1.995306 -0.885632 1.630176
H 1.294273 -1.960028 0.796190
H -0.281017 -0.996864 2.027550
H -1.688099 -1.145531 1.369931
H -1.170727 0.820093 1.116127
H -0.924585 1.042488 -0.393586
H 0.441708 2.452059 0.427252
H 1.438987 2.056908 -0.652936
H -1.747975 -1.323907 -0.840743
H -3.112990 -0.807038 -0.355438
O -3.875228 1.118517 -0.147795
H -2.943435 1.408870 0.093895

NO3-.OH.(H2O)7
H=-891.440974
O 2.671363 0.373746 -0.566346

O 2.024504 -0.890702 1.719127
O 0.990857 -3.192343 0.388161
O -0.239847 0.549740 2.294243
O -0.214354 2.332202 0.077815
O -2.660086 -0.519512 1.381358
O -1.330859 -1.763304 -0.851919
N -0.548900 -0.947007 -1.387351
O -0.926012 0.228392 -1.638471
O 0.623281 -1.296374 -1.682432
O -2.984218 1.730065 -0.291803
H -2.971034 0.306532 0.961645
H -2.332494 -1.038633 0.626836
H 0.036756 -3.198907 0.532993
H 1.049012 -2.708893 -0.457021
H 2.028159 -0.050705 -1.158536
H 2.521325 -0.076516 0.309282
H 1.212447 -0.417798 2.018708
H 1.719367 -1.766441 1.415342
H -1.096085 0.105221 2.119478
H -0.202920 1.262498 1.628650
H -0.232068 1.627651 -0.594785
H 0.654583 2.765097 -0.032830
H -2.249454 2.297216 -0.007508
H -2.551938 1.151896 -0.943026
H 2.624109 1.989136 -0.460012
O 2.499438 2.995803 -0.360705

NO3-.OH.(H2O)8
H=-967.900279
O 3.638760 0.288663 -1.110001
O 1.649715 1.727203 0.264167
O 1.465629 -0.067145 2.297643
O -0.665123 3.214079 0.122104
O 0.815159 0.199011 -1.855348
O 2.823889 -1.810001 0.606357
O -1.562528 1.654523 -1.994369
O 0.259796 -2.236871 -0.556230
O -1.142185 -1.025014 1.483860
N -1.854811 -0.083498 1.025208
O -1.915835 1.000899 1.609344
O -2.506216 -0.279660 -0.046898
H 0.540919 -0.363592 2.279971
H 1.976223 -0.767052 1.831476
H 2.078957 -2.180602 0.105019
H 3.230856 -1.147881 0.009001
H 2.967419 0.133958 -1.792861
H 3.221041 0.998116 -0.586784
H 1.071510 0.733317 -1.065596
H 0.609875 -0.688183 -1.499209
H -0.207412 -1.862432 0.221561
H -0.441621 -2.734303 -1.005949
H -0.748758 1.124239 -2.135014
H -2.110067 1.085027 -1.429900
H -0.990965 2.828168 -0.722496
H -1.175076 2.727082 0.786192
H 0.934929 2.403840 0.278008
H 1.562033 1.189931 1.089308
O -2.486412 -2.789315 -1.174589
H -2.533374 -1.906113 -0.707497

NO3-.OH.(H2O)9
H=-1044.354728
O -3.186346 -1.630122 -0.708084
O -2.998809 0.887761 -1.837466
O -2.704244 1.997366 0.700828
O -2.505588 -0.553946 1.744368
O -0.381710 -2.163133 -0.860040
O 2.327644 -2.508164 -1.402290
O -0.115058 0.485928 -1.567974
O 0.124939 2.294574 0.529749
O 1.295775 0.434173 2.240225
N 1.304814 -0.671261 1.665932
O 2.315799 -1.021534 0.989314
O 0.317160 -1.444219 1.748134
O 2.603147 0.539962 -1.342825
H -3.029717 -1.339715 0.220337
H -2.344083 -2.040210 -0.959570
H -1.573206 -0.717518 1.951756
H -2.576636 0.397589 1.499918
H -0.199243 -2.113356 0.098475
H 0.464454 -2.464881 -1.249153
H -3.188432 -0.028665 -1.527658
H -2.056711 0.856126 -2.068419
H -0.332009 -0.432956 -1.289115
H -0.206870 1.063862 -0.780865
H -1.800419 2.348616 0.659383
H -2.907788 1.717063 -0.220137
H 0.487024 1.768723 1.267678
H 0.879715 2.792516 0.164486
H 1.640047 0.484357 -1.563207
H 2.667476 0.122895 -0.462008
H 2.606001 -1.700474 -1.856883
H 2.403573 -2.259431 -0.462376
O 2.561370 3.183392 -0.741194
H 2.700515 2.228001 -1.026131

NO3-.OH.(H2O)10
H=-1120.812001
O -2.600806 -2.575275 0.074948
O -3.492722 -0.653683 -1.712448
O -3.676309 1.201454 0.342097
O -2.441007 -0.607054 2.021050
O 0.191211 -2.100620 -0.329626
O 2.566076 -2.011314 -1.689361
O 1.946122 0.749185 -1.976721
O -0.702724 0.130010 -1.706164

O -1.088906 2.335460 -0.076319
O 1.537286 -0.772706 1.857958
N 1.237865 0.421419 1.586102
O 2.035580 1.116122 0.904018
O 0.157953 0.900001 1.987825
O 1.364394 3.380934 -0.866246
H -2.562688 -1.976903 0.854499
H -1.668928 -2.685974 -0.172392
H -1.560648 -0.246599 2.195908
H -2.923917 0.095806 1.525658
H 0.491520 -1.878928 0.565480
H 1.018525 -2.215751 -0.857720
H -3.292911 -1.433287 -1.144623
H -2.615473 -0.386758 -2.033626
H -0.532419 -0.657406 -1.143716
H -0.949932 0.865475 -1.103342
H -2.968262 1.822143 0.108822
H -3.744819 0.595400 -0.431672
H -0.700990 1.999559 0.752736
H -0.387438 2.904741 -0.455678
H 0.979707 0.550724 -2.036037
H 2.118759 0.753858 -1.020657
H 1.612995 2.753639 -1.566707
H 1.707145 2.948699 -0.068535
H 2.475344 -1.107403 -2.047607
H 3.213053 -1.917929 -0.967917
H 3.179422 -1.109031 1.213145
O 4.009715 -1.420515 0.760397

NO3-.OH.(H2O)11
H=-1197.264705
O 4.413440 -0.954896 0.267965
O 2.105568 -2.464352 0.666035
O 0.215004 -0.870544 1.616938
O 1.242094 -2.196696 -1.957158
O -1.145692 -1.304707 -0.797847
O -2.420994 1.134961 -1.092970
O -2.655630 1.720408 1.489536
O -3.656941 -2.079200 0.182835
O -2.529385 -0.817586 2.427915
O 3.086016 0.816701 1.989710
O 2.000862 1.952519 -0.358579
N 0.740174 2.094093 -0.291946
O 0.100812 2.446807 -1.294000
O 0.174034 1.863432 0.806353
O 2.991262 0.092463 -2.054395
H 4.107519 -0.290840 0.918921
H 4.116345 -0.615001 -0.592170
H 2.647058 1.344457 1.294580
H 2.375295 0.319764 2.411748
H 2.602827 0.852296 -1.563977
H 3.293659 0.443607 -2.897153
H 3.003749 -2.075676 0.598322
H 1.816822 -2.562212 -0.268753
H 1.773505 -1.396608 -2.108736
H 0.341897 -1.883611 -1.737456
H 0.403594 0.054642 1.389074
H 0.986113 -1.444273 1.334344
H -0.726600 -1.206147 0.083903
H -1.554538 -0.429551 -0.978471
H -3.010459 -1.324023 1.739132
H -1.586322 -1.021377 2.297168
H -1.695424 1.731151 -1.349731
H -2.606168 1.362172 -0.136828
H -2.666816 0.834911 1.936107
H -1.733135 2.011988 1.534505
H -2.791165 -2.046787 -0.270040
H -4.220117 -1.500846 -0.355838
O -4.693735 -0.120554 -1.833358
H -3.872564 0.433255 -1.706879
